# Concordance of multigene genealogy along with morphological evidence unveils five novel species and two new records of boletoid mushrooms (fungi) from India

**DOI:** 10.1038/s41598-024-59781-2

**Published:** 2024-04-23

**Authors:** Kanad Das, Aniket Ghosh, Sudeshna Datta, Upendra Singh, Dyutiparna Chakraborty, Debala Tudu, Alfredo Vizzini

**Affiliations:** 1 Central National Herbarium, Botanical Survey of India, P.O.-B. Garden, Howrah, 711103 India; 2 Department of Botany and Microbiology, H.N.B. Garhwal University, Srinagar Garhwal, Uttarakhand 246174 India; 3 Eastern Regional Centre, Botanical Survey of India, Shillong, Meghalaya 793003 India; 4 Department of Life Sciences and Systems Biology, University of Torino, 10125 Turin, Italy

**Keywords:** Agaricomycetes, Biodiversity, Boletaceae, Morphology, Multigene phylogeny, Novel species, Ecology, Evolution, Molecular biology, Plant sciences

## Abstract

Agaricales, Russulales and Boletales are dominant orders among the wild mushrooms in Basidiomycota. Boletaceae, one of the major functional elements in terrestrial ecosystem and mostly represented by ectomycorrhizal symbionts of trees in Indian Himalaya and adjoining hills, are extraordinarily diverse and represented by numerous genera and species which are unexplored or poorly known. Therefore, their hidden diversity is yet to be revealed. Extensive macrofungal exploration by the authors to different parts of Himalaya and surroundings, followed by through morphological studies and multigene molecular phylogeny lead to the discovery of five new species of wild mushrooms: *Leccinellum bothii* sp. nov., *Phylloporus himalayanus* sp. nov., *Phylloporus smithii* sp. nov., *Porphyrellus uttarakhandae* sp. nov., and *Retiboletus pseudoater* sp. nov. Present communication deals with morphological details coupled with illustrations and phylogenetic inferences. Besides, *Leccinellum sinoaurantiacum* and *Xerocomus rugosellus* are also reported for the first time from this country.

## Introduction

The family Boletaceae (Basidiomycota, Boletales) represents mushrooms (macrofungi) that are mainly characterized by soft, fleshy, pileate, centrally stipitate and tubulose to rarely lamellate or loculate hymenophore^1,2^. Being ectomycorrhizal associates of angiospermous and gymnospermous trees (*Quercus*, *Lithocarpus*, *Castanopsis*, *Betula*, *Shorea*, *Abies*, *Pinus*, *Picea*, *Larix*, *Tsuga*, etc.) ^1,3^ they are key components of terrestrial ecosystems and one of the dominant wild mushrooms in Indian Himalaya. However, due to complex and overlapping morphological features among its genera and the limited phylogenetic information kept this important mushroom family unresolved for many years in terms of its systematics and evolution. About 50 genera and 800 species were recognised in this family by the Dictionary of Fungi^4^. Morphotaxonomy, when used alone or combined with molecular phylogenetic analyses using less informative ribosomal genetic markers such as LSU, SSU etc., failed to resolve several issues. Several genera remained polyphyletic, delimitation among many genera was obscured, and evolutionary relationships remained unclear. However, over the last decade, a combined approach, utilizing multilocus molecular phylogeny alongside morphology, revealed crucial insights. Three protein-coding genes, namely *rpb1*(RNA polymerase II largest subunit), *rpb2* (RNA polymerase II second largest subunit), and *tef* 1-α (translation elongation factor 1α), played the key role to give the proper phylogenetic framework for Boletaceae^2^. These revolutionary changes lead to the discovery of more than 100 genera and *ca* 1200 species^5^ from the world. Moreover, this mode of investigation redefined seven major clades within this family, namely, subfamilies Austroboletoideae, Boletoideae, Chalciporoideae, Leccinoideae, Xerocomoideae, Zangioideae, and *Pulveroboletus* group^2^.

In subtropical to subalpine forests of India, the three major mushroom-producing orders are Agaricales Underw., Russulales Kreisel ex P.M. Kirk, P.F. Cannon & J.C. David, and Boletales E.-J. Gilbert (Basidiomycota). Himalaya and adjacent hilly ranges, the home (type locality) of numerous wild mushrooms, are still unexplored to poorly explored. Hidden diversity is much awaited. Boletaceae is no exception of it. Presently, 85 species belonging to 24 genera are known from Indian Himalaya^5–8^. Recently, in the month of August (2023), the authors have taken macrofungal exploration to three districts (Rudraprayag, Chamoli and Bageshwar) of the state Uttarakhand in western Himalaya and East Khasi Hills of Meghalaya in Northeast India. Intensive surveys were undertaken to four forested areas namely, Baniyakund: temperate mixed (broadleaf and coniferous) forest in Rudraprayag district (Uttarakhand), Didna: temperate broadleaf forest in Chamoli district (Uttarakhand), Dhakuri: temperate to subalpine mixed forest in Bageshwar district (Uttarakhand) and Sohra, sub-temperate broad leaf forests in East Khasi Hills District (Meghalaya). A large number of boletoid mushrooms were collected. Thorough observation of morphological features followed by a multigene molecular phylogeny using ITS, LSU, *rpb2* and/or *tef* 1-α markers uncovered five novel species and two first records in Boletaceae from this country. *Leccinellum bothii* sp. nov., *Phylloporus himalayanus* sp. nov., *Phylloporus smithii* sp. nov., *Porphyrellus uttarakhandae* sp. nov., *Retiboletus pseudoater* sp. nov. are proposed herein. Moreover, *Leccinellum sinoaurantiacum* (M. Zang & R.H. Petersen) Yan C. Li & Zhu L. Yang and *Xerocomus rugosellus* (W.F. Chiu) F.L. Tai which were known earlier from China are also recorded for the first time from India.

## Results

### Phylogenetic inferences

**Figure 1:**
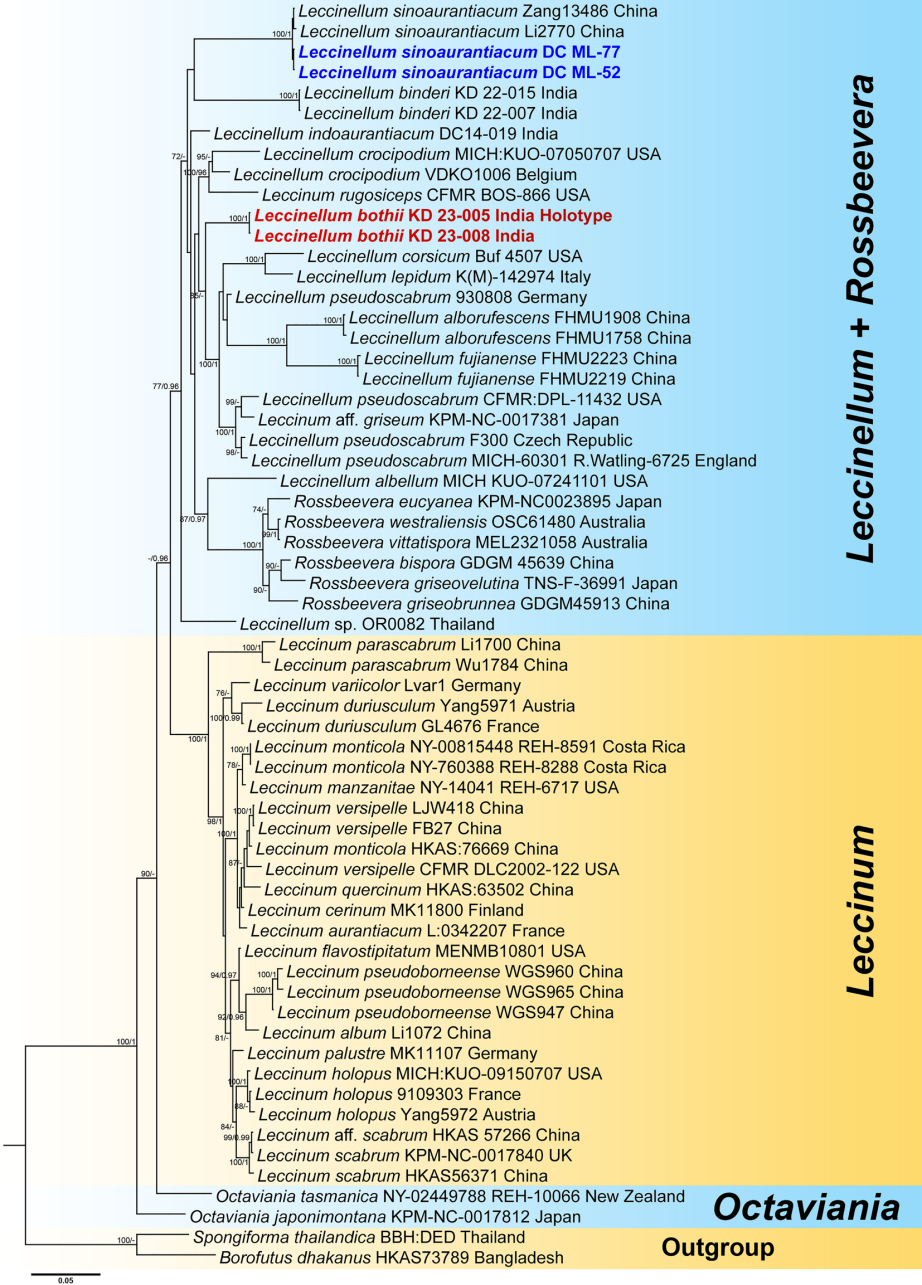
Phylogram generated by Bayesian analysis based on combined sequence data of LSU, *rpb2* and *tef* 1-α for *Leccinellum bothii*,* L*. *sinoaurantiacum* and allied species. Maximum likelihood bootstrap support values (MLbs) ≥ 70% are shown on the left of “/” and Bayesian posterior probabilities (BPP) ≥ 0.95 are shown on the right above or below the branches at nodes. *Leccinellum bothii* and *L*. *sinoaurantiacum* are placed in bold red and blue font respectively to highlight their phylogenetic positions in the tree.

In our present study, the three-locus dataset (LSU + *rpb2* + *tef* 1-α) of *Leccinellum* consisted of 62 taxa and 2,311 nucleotide sites, including gaps. *Borofutus dhakanus* Hosen & Zhu L. Yang and *Spongiforma thailandica* Desjardin, Manfr. Binder, Roekring & Flegel were selected as outgroup taxa. Phylogenetic analysis revealed that sequences from our first species, *Leccinellum bothii* (voucher nos. KD 23-005 and KD 23-008) clustered with the species of *L. crocicum* (voucher no. Buff 4507), *L. lepidium* (voucher no. K(M)-142974), *L. fujianense* (voucher nos. FHMU2219 & FHMU2223), *L*. *alborufescens* (voucher nos. FHMU1908 & FHMU1758), *L*. aff. *griseum* (voucher no. KPM-NC-0017381) and *L*. *pseudoscabrum* (voucher no. CFMR:DPL-11432, 930808, F300 & MICH-60301 R.Watling-6725) with moderate support (MLbs = 85%), forming a distinct clade within the *Leccinellum* lineage. However, our specimens were recovered as distinct species within the phylogenetic tree (Fig. 1). Conversely, our second species, *Leccinellum sinoaurantiacum* (voucher nos. DC ML-52 and DC ML-77) is nested within the *L. sinoaurantiacum* clade consisting of sample vouchers (Li2770 and Zang13486) collected from China and suggesting its strong similarity or conspecificity with the Asian species of *L. sinoaurantiacum* with a strong (MLbs = 100%, BPP = 1) support (Fig. 1).

**Figure 2:**
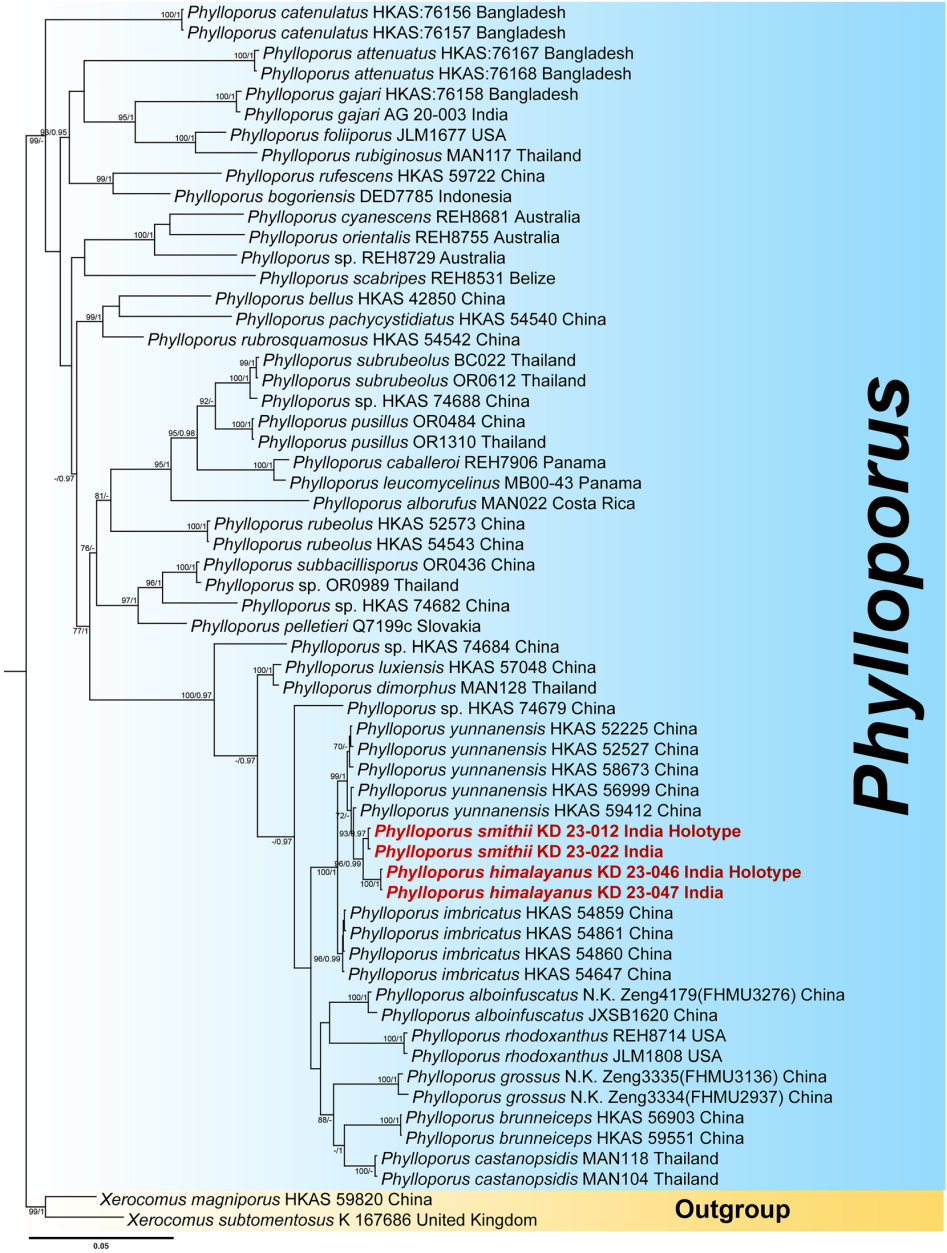
Phylogram generated by Bayesian analysis based on combined sequence data of ITS, LSU and *tef* 1-α for *Phylloporus himalayanus*, *P. smithii* and allied species. Maximum likelihood bootstrap support values (MLbs) ≥ 70% are shown on the left of “/” and Bayesian posterior probabilities (BPP) ≥ 0.95 are shown on the right above or below the branches at nodes. *Phylloporus himalayanus* and *P. smithii* are placed in bold red font to highlight their phylogenetic positions in the tree.

Again, the three-locus dataset (ITS + LSU + *tef* 1-α) for *Phylloporus* comprised of 60 taxa and 2400 nucleotide sites, including gaps. *Xerocomus magniporus* M. Zang & R.H. Petersen and *X*. *subtomentosus* (L.) Quél. were selected as outgroup taxa following. In the phylogram, sequences from our third and fourth species, *Phylloporus himalayanus* (voucher nos. KD 24-046 and KD 23-047) and *P*. *smithii* (voucher nos. KD 22-012 and KD 22-022), clustered with the *P*. *yunnanensis* clade with strong support (MLbs = 98%, BPP = 0.96), being sister to the *P*. *imbricatus* clade. However, our two species were identified as distinct novel taxa within the phylogenetic tree (Fig. 2).

**Figure 3:**
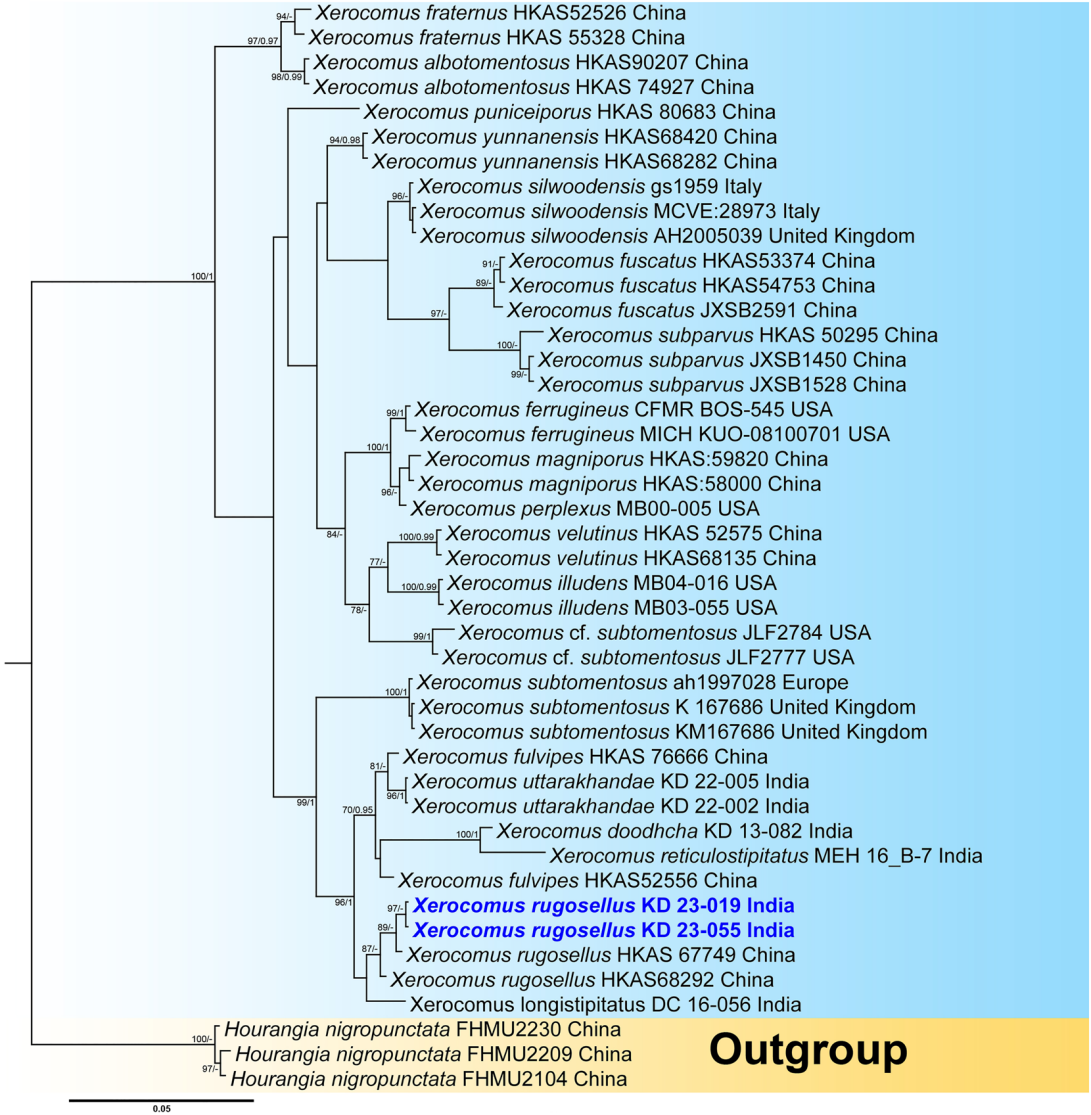
Phylogram generated by Bayesian analysis based on combined sequence data of ITS and LSU for *Xerocomus rugosellus* and allied species. Maximum likelihood bootstrap support values (MLbs) ≥ 70% are shown on the left of “/” and Bayesian posterior probabilities (BPP) ≥ 0.95 are shown on the right above or below the branches at nodes. *Xerocomus rugosellus* is placed in bold blue font to highlight its phylogenetic position in the tree.

The two-locus (ITS + LSU) dataset of *Xerocomus*, comprising 44 taxa and 1393 nucleotide sites, including gaps, used *Hourangia nigropunctata* (W.F. Chiu) Xue T. Zhu & Zhu L. Yang as outgroup taxa following. The combined (ITS + LSU) phylogenetic analysis showed that the two collections of our fifth species, *Xerocomus rugosellus* (voucher nos. KD 23-019 and KD 23-055) is nested within the *X. rugosellus* clade, consisting of sample vouchers (HKAS 67749 and HKAS68292) collected from China and suggesting its strong similarity or conspecificity with the Asian species of *X. rugosellus* with a strong (MLbs = 87%) support. (Fig. 3).

**Figure 4:**
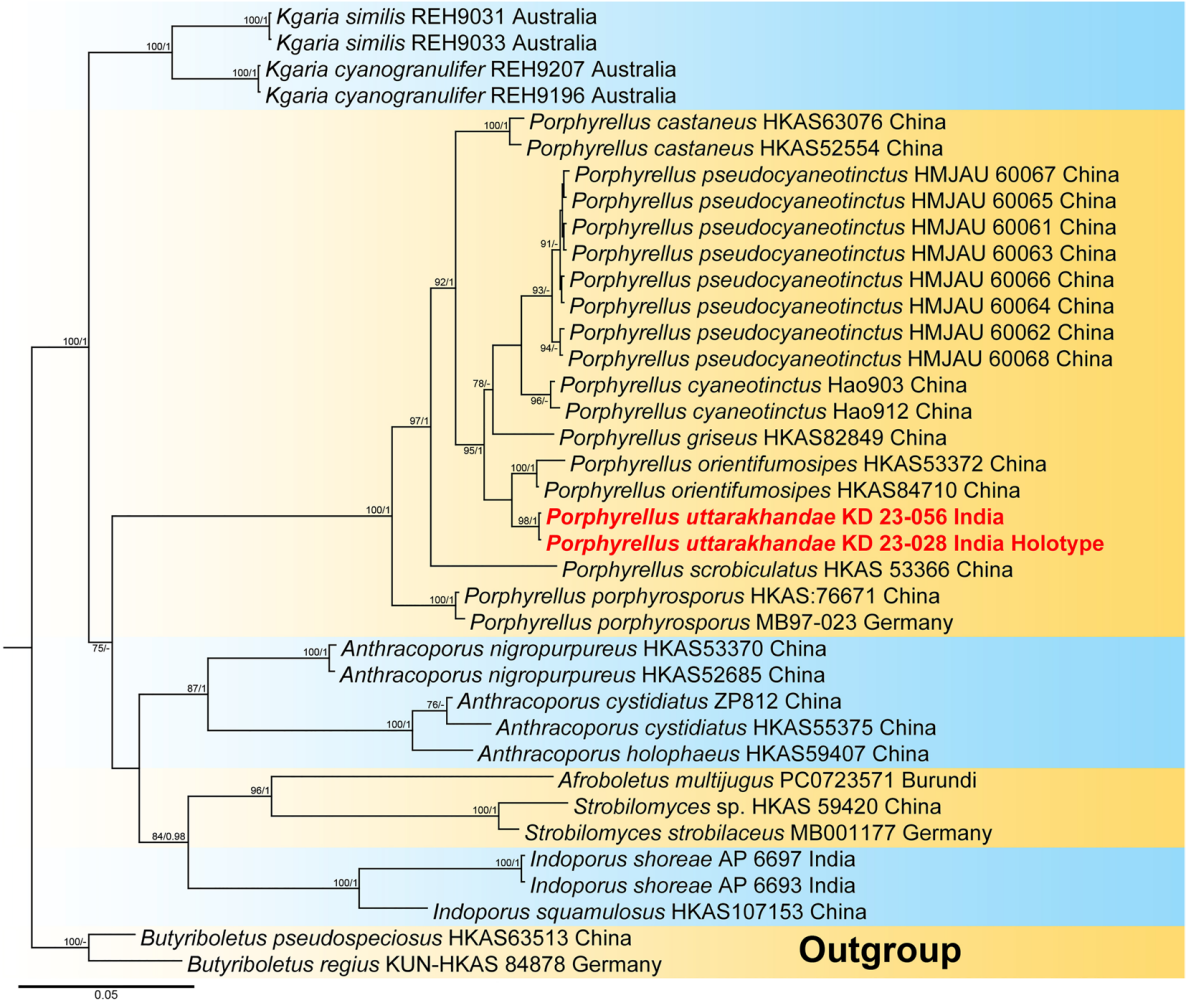
Phylogram generated by Bayesian analysis based on combined sequence data of LSU, *rpb2* and *tef* 1-α for *Porphyrellus uttarakhandae* and allied species. Maximum likelihood bootstrap support values (MLbs) ≥ 70% are shown on the left of “/” and Bayesian posterior probabilities (BPP) ≥ 0.95 are shown on the right above or below the branches at nodes. *Porphyrellus uttarakhandae* is placed in bold red font to highlight its phylogenetic position in the tree.

Three-locus dataset (LSU + *rpb2* + *tef* 1-α) of *Porphyrellus*, comprised of 37 taxa and 1980 nucleotide sites, including gaps. *Butyriboletus pseudospeciosus* Kuan Zhao & Zhu L. Yang and *B*. *regius* (Krombh.) D. Arora & J.L. Frank were selected as outgroup taxa following. Combined three-locus phylogenetic analyses revealed that two collections of our sixth species, *Porphyrellus uttarakhandae* (voucher nos. KD 23-028 and KD 23-056), clustered with *Por. orientifumosipes* (voucher nos. HKAS84710 and HKAS53372) from China without a strong support, being sister to the *Por. pseudocyaneotinctus* and *Por. griseus* clade. However, our specimens were recovered as distinct species within the phylogenetic tree (Fig. 4).

**Figure 5:**
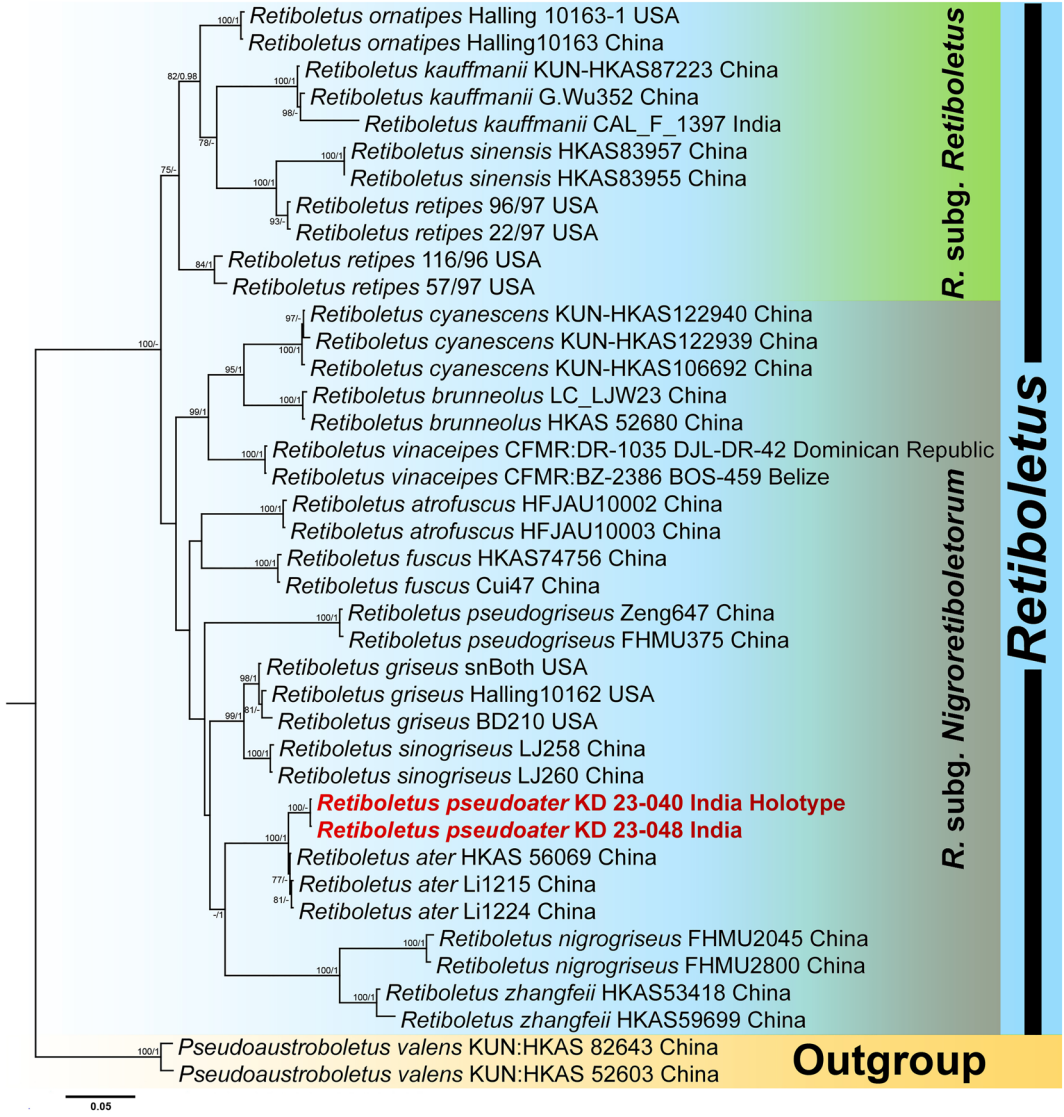
Phylogram generated by Bayesian analysis based on combined sequence data of ITS, LSU and *tef* 1-α for *Retiboletus pseudoater* and allied species. Maximum likelihood bootstrap support values (MLbs) ≥ 70% are shown on the left of “/” and Bayesian posterior probabilities (BPP) ≥ 0.95 are shown on the right above or below the branches at nodes. *Retiboletus pseudoater* is placed in bold red font to highlight its phylogenetic position in the tree.

On the other hand, the three-locus dataset (ITS + LSU + *tef* 1-α) of *Retiboletus* consisted of 40 taxa and 1999 nucleotide sites, including gaps. *Pseudoaustroboletus valens* (Corner) Yan C. Li & Zhu L. Yang was used as outgroup taxa following. Combined three-locus phylogenetic analyses revealed that two collections of our seventh species, *Retiboletus pseudoater* (voucher nos. KD 23-040 and KD 23-048), nested with *R*. *ater* (voucher nos. Li1215, Li1224 and HKAS 56069) from China with strong support (MLbs = 100%, BPP = 1). However, our specimens were identified as distinct species within the phylogenetic tree (Fig. 5).

### Taxonomy

#### Leccinoideae

**Figure 6:**
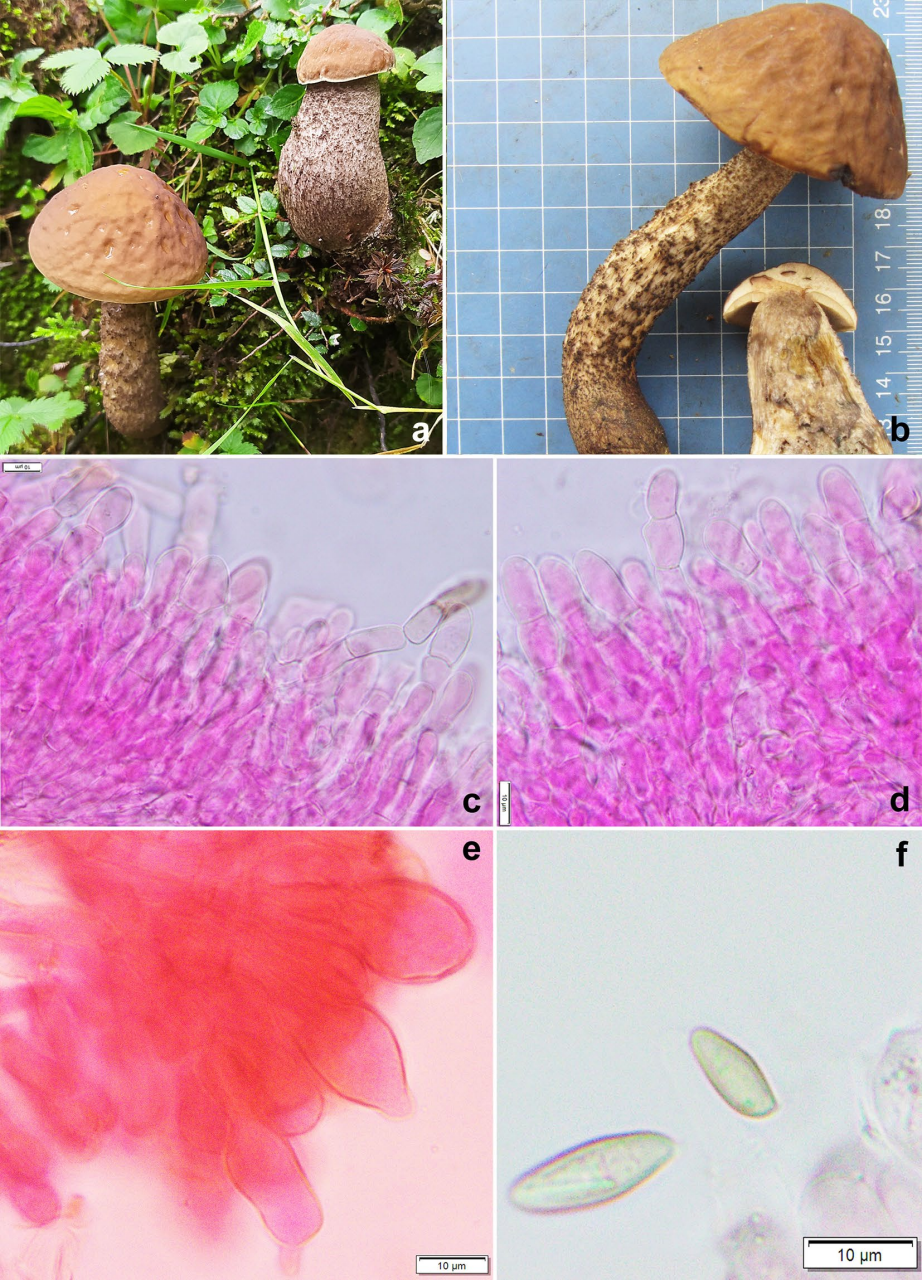
*Leccinellum bothii*. (KD 23–005, holotype). (**a**,**b**) Fresh basidiomata. (**c**,**d**) Pileipellis. (**e**) Caulocystidia. (**f**) Basidiospores. Scale bars: (**c**–**f**) = 10 µm.

**Figure 7:**
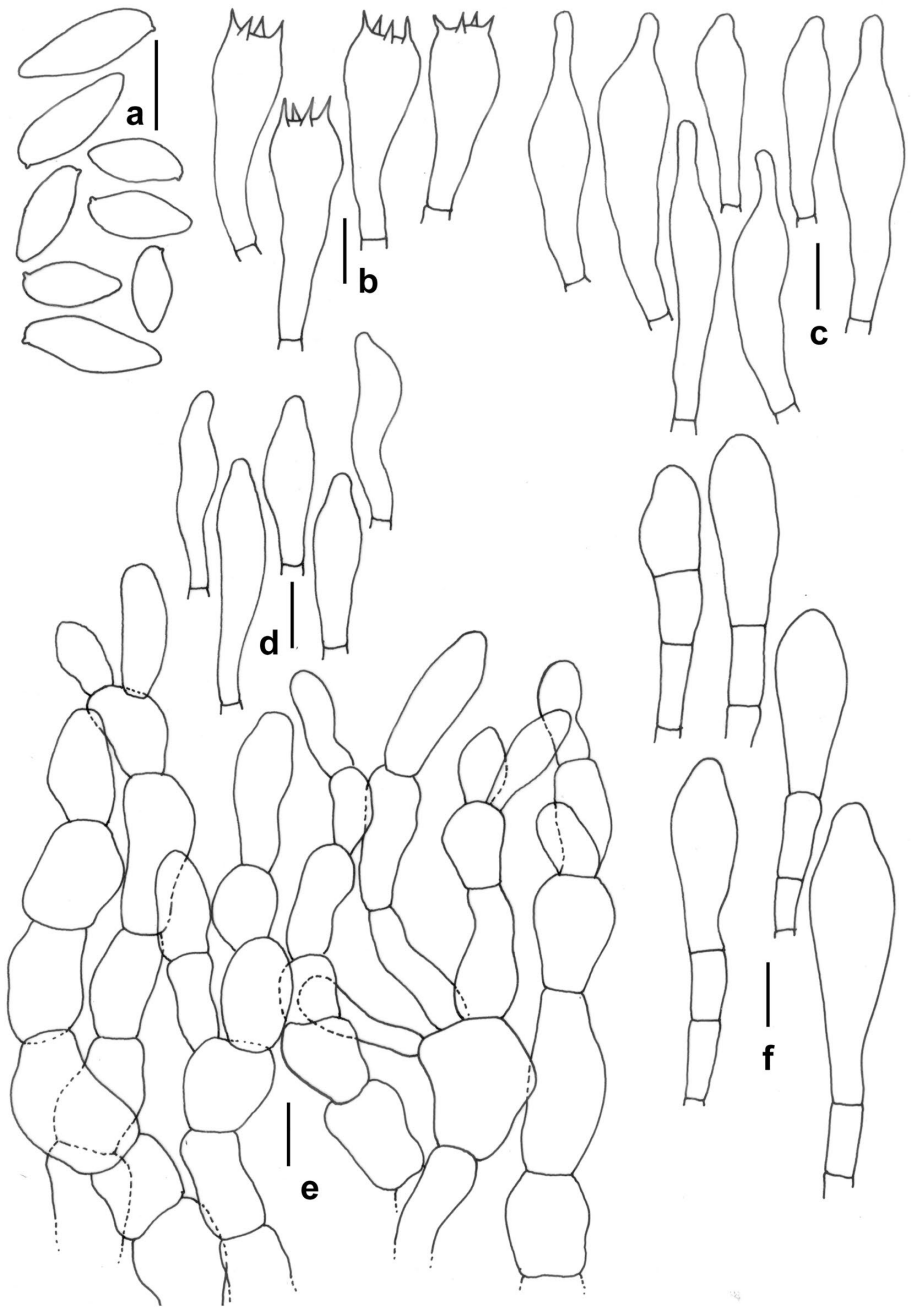
*Leccinellum bothii*. (KD 23-005, holotype). (**a**) Basidiospores. (**b**) Basidia. (**c**) Pleurocystidia. (**d**) Cheilocystidia. (**e**) Elements of pileipellis. (**f**) Cheilocystidia. Scale bars: (**a**–**f**) = 10 µm.

*Leccinellum bothii* K. Das, A. Ghosh, Sudeshna Datta, U. Singh & Vizzini sp. nov. *Mycobank*: MB 851128. *Holotype* INDIA, Uttarakhand, Rudraprayag district, Baniyakund, 30° 29.000′ N 79° 10.743′ E, alt. 2622 m, temperate mixed forests, under *Quercus* sp., 3 August 2023, *K. Das*, KD 23-005 (CAL 1953, holotype!) (Figs. 1, 6, 7).

*Etymology* Commemorating E.E. Both for his important contribution to the systematics of Boletaceae.

*Diagnosis* Distinguished from other allied species of *Leccinellum* by a rugulose or pitted, brown to dark brown pileus, a brown to greyish orange colour changes of hymenophore, unchanging pileus context, greyish black (with greyish orange near base) colour changes of stipe context, pileipellis composed of chains of elongate, subglobose to pyriform elements, the occurrence in temperate Himalaya and LSU, *rpb2*, and *tef* 1-α sequence data.

*Basidiomata* small to medium-sized. *Pileus* 29–54 mm in diam., hemispherical to conic or convex; surface somewhat rugulose to pitted, non-viscid, with a narrow flap of tissue at margin, caramel brown to brownish orange (6C4–5), yellowish white (3A2) or paler near margins, mostly unchanging with maturity; turning reddish brown (8D7) with KOH and greenish with FeSO_4_. *Hymenophore* slightly depressed near stipe apex, adnexed; pore surface yellowish white (3A2) to yellow, becoming brown, greyish orange (5B3) then linoleum brown (5E7) with maturity or on bruising; pores are rounded, 2–3/mm. *Tubes* adnexed, 2–6 mm long, yellowish white to pale yellow (2–3A2–3), slowly becoming brownish on exposure. *Stipe* 60–98 × 18–30 mm, clavate when young, more or less cylindrical with tapering apex and a swollen base; surface with striations near apex, distinctively scabrous up to middle or slightly below, yellowish white to pale yellow (3A2–3) with brown (6D6) to dark brown (7F8) squamules that becomes grey black to black when bruised, becoming pale orange towards base on handling. *Context* in pileus, up to 8 mm thick, white to yellowish white, unchanging; context in stipe, chalky, slowly becoming yellowish then greyish black on exposure, greyish orange (5B4) near base; turning dull green (25E4), then slowly even darker with FeSO_4_ and pale yellow (3A3) with KOH. *Basal mycelium* white. *Taste* mild and *odour* indistinct. *Spore print* not obtained.

*Basidiospores* 8.0–12.2–16.7 × 4.5–5.1–6.0 µm, (n = 30, Q = 1.78–2.41–3.2), subfusoid to elongate and inequilateral in side view with distinct suprahilar depression, light yellow, smooth, inamyloid. *Basidia* 29–34 × 11–12 µm, clavate, 4-spored; sterigmata 3–5 × 0.5–1 µm. *Pleurocystidia* 31–49 × 7–11 µm, abundant, fusoid-ventricose with subcapitate to appendiculate apex, thin-walled, hyaline, emergent up to 20 µm. *Tube edge* fertile. *Cheilocystidia* 26–39 × 9–12 µm, abundant, fusoid-ventricose with rounded to subcapitate apex, thin-walled, hyaline. *Hymenophoral trama* divergent, hyphae cylindrical, septate, 3–6 µm wide. *Pileipellis* 100–120 µm thick, a trichodermium, composed of branched chains of subcylindric, subglobose, clavate to pyriform elements; terminal elements 7–26 × 5–11 µm, cylindrical to clavate, with brown intracellular pigmentation, thromboplerous hyphae present. *Stipitipellis* up to 100 µm thick, a trichodermium, composed of loosely arranged, erect, branched, septate hyphae, terminal elements 23–40 × 5–9 µm, clavate to cylindric; with frequent clusters of basidia and cystidia (caulohymenium); *caulocystidia* 41–53 × 9–13 µm, clavate, pyriform, ventricose; *caulobasidia* 31–34 × 9–11 µm, narrowly clavate, 4-spored. *Clamp connections* absent in all tissues.

*Additional specimen examined*: INDIA, Uttarakhand, Rudraprayag District, Baniyakund, 30° 28.892′ N 79° 10.761′ E, alt. 2585 m, temperate mixed forests under *Quercus* sp., 3 August 2023, *K. Das*, KD 23-008 (CAL 1954).

**Figure 8:**
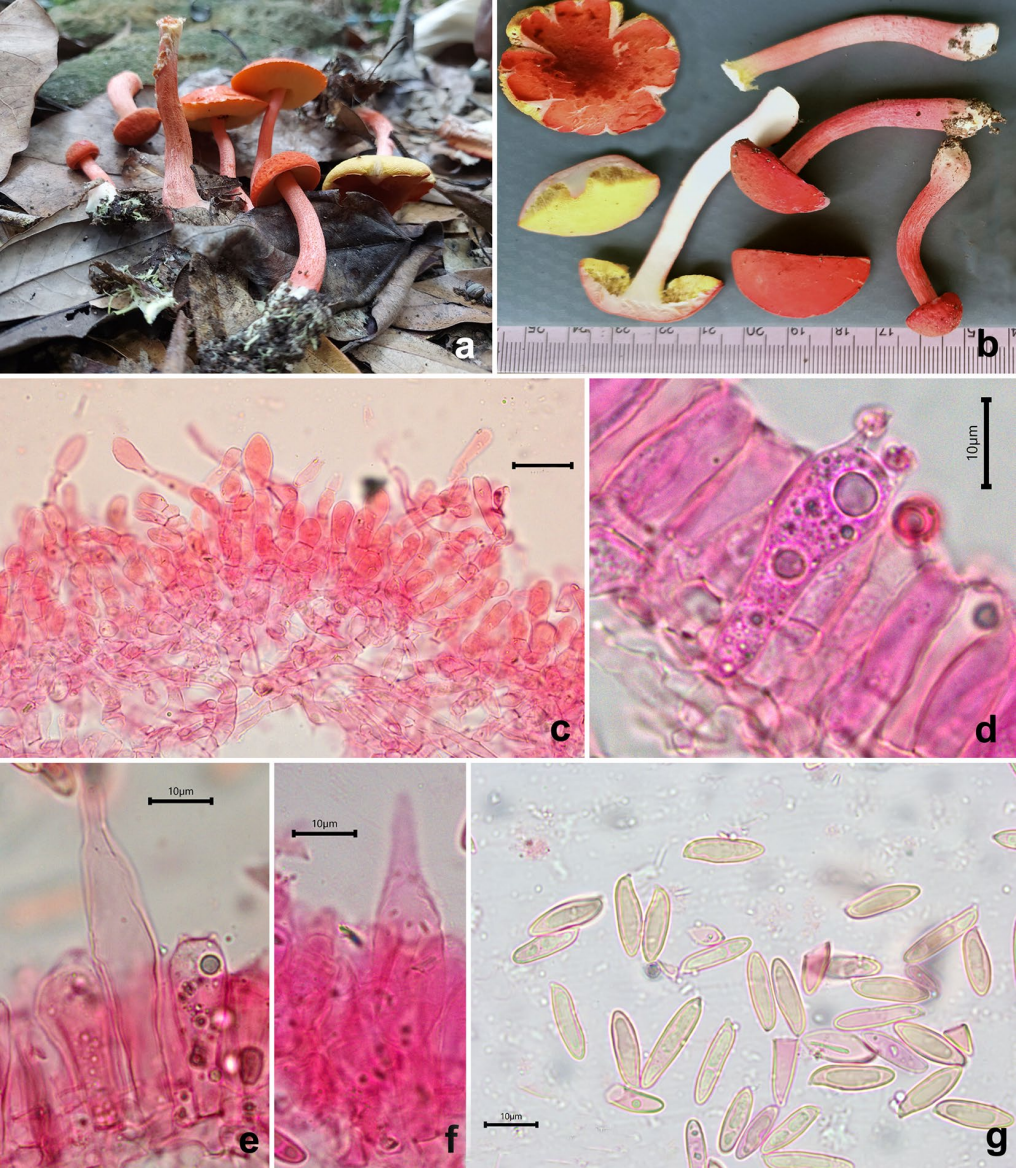
*Leccinellum sinoaurantiacum* (DC ML-52). (**a**,**b**) Fresh basidiomata. (**c**) Pileipellis. (**d**) Basidia. (**e**,**f**) Pleurocystidia. (**g**) Basidiospores. Scale bars: (**c**) = 25 μm, (**d**–**g**) = 10 μm.

**Figure 9:**
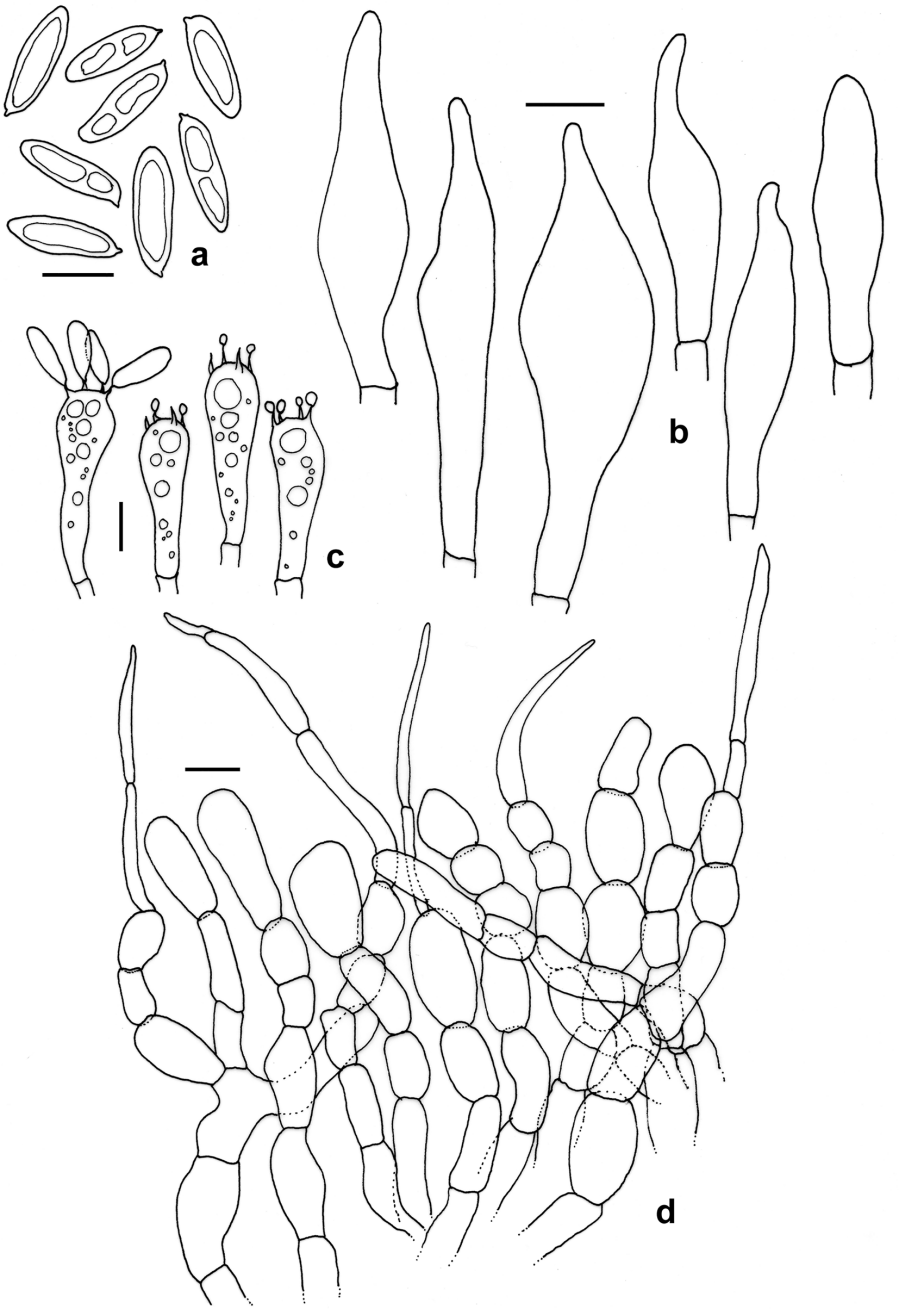
*Leccinellum sinoaurantiacum* (DC ML-52). (**a**) Basidiospores. (**b**) Pleuro- and cheilocystidia. (**c**) Basidia. (**d**) Pileipellis. Scale bars: (**a**–**d**) = 10 μm.

*Leccinellum sinoaurantiacum* (M. Zang & R.H. Petersen) Yan C. Li & Zhu L. Yang, *The Boletes of China: Tylopilus s.l.* (Singapore): 164 (2021) (Figs. 1, 8, 9).

*Basidiomata* small to medium-sized. *Pileus* 10–40 mm in diam., hemispherical to convex rarely applanate; surface moist, gelatinous when wet, scarlet or crimson red, vivid red (10A8) when young, with maturity orange-red (8A7); turning brownish red (8C7) with KOH. *Hymenophore* depressed near stipe apex, adnate; pore surface light yellow to yellow (1–2A5–6) no change on bruising; pores angular, 1–1.4/mm. *Tubes* adnate, 8 mm long, yellow (2A4), unchanging on exposure. *Stipe* 40–70 × 5–10 mm, more or less cylindrical with tapering apex and a broader base; surface with squamules, denser towards base, pink to scarlet red (10A5–6). *Context* in pileus up to 5 mm thick, cream yellow to pale pink, unchanging on exposure; context in stipe, solid, cream white to pale pink. *Basal mycelium* yellow. *Taste mild* and *odour* fungoid. *Spore print* salmon pink.

*Basidiospores* 14.8–17.5–20 × 3.6–4.5–5.5 [Q = 2.9–3.2–3.8], elongated, light yellow, smooth, inamyloid. *Basidia* 32–40 × 11–12 µm, clavate, 4-spored. *Pleurocystidia* 43–65 × 11–19 µm, less in number, fusiform to subfusiform and ventricose, appendiculate apex, thin-walled, emergent up to 22 µm. *Tube edge* fertile. *Cheilocystidia* same as pleurocystidia. *Hymenophoral trama* divergent, hyphae cylindrical, septate, 6–9 µm wide. *Pileipellis* 120–130 µm thick, an ixohyphoepithelium, composed of two layers; upper layer composed of erect to suberect septate filamentous hyphae submerged under a gluten layer; lower layer composed of branched chains of subcylindric to subglobose or globose elements; terminal elements of upper layer 11–25 × 8–12 µm. *Stipitipellis* up to 100 µm thick, a trichodermium, with clusters of basidia and cystidia (caulohymenium); *caulocystidia* 30–50 × 9–19 µm, clavate, pyriform, ventricose; *caulobasidia* 29–36 × 6–12 µm, narrowly to broadly clavate, 4-spored. *Clamp connections* absent in all tissues.

*Specimens examined* INDIA, Meghalaya, East Khasi Hills district, Sohra, 25° 18.736′ N 91° 45.926′ E, alt. 1535 m, sub-temperate broad leaf forests under *Castanopsis* sp., 8 August 2023, *D. Chakraborty* and *D. Tudu*, DC ML-52 (ASSAM F001); ibid.*,* Mawlyndiar, 25° 18.641′ N 91° 45.321′ E, alt. 1535 m, sub-temperate broad leaf forests under *Castanopsis* sp., 8 August 2023, *D. Chakraborty*, DC ML-77 (ASSAM F002).

*Notes* Presence of yellow pore surface, a distinctively scaly stipe surface and a trichodermium (or rarely ixohyphoepithelium) pattern of the pileipellis undoubtedly place these two species under the genus *Leccinellum* Bresinsky & Manfr. Binder^9^. In the field, our proposed new species, *Leccinellum bothii* is quite similar to *L. alborufescens* N.K. Zeng, R. Xue & S. Jiang and *L. fujianense* N.K. Zeng, R. Xue & Zhi Q. Liang (both are originally described from China). However, both *L. alborufescens* and *L. fujianense* can be differentiated from the present species by showing the change in the overall colour of stipe surface to red (in *L. bothii*, never changes to red except at base that becomes pale orange), pileus and stipe context to red (in *L. bothii*, pileus context remains unchanged, stipe context changes to greyish black except near base that changes to greyish orange). Additionally, *L*. *alborufescens* and *L*. *fujianense* have distinctively smaller basidiospores and are known to occur in tropical and subtropical forests, respectively, whereas *L*. *bothii* is found in temperate mixed forests^10^. Further, *L. binderi* K. Das, A. Ghosh & Vizzini, another recently discovered species from the same locality easily falls apart from *L. bothii* by differently looking pileus (hemispherical to convex to applanate pileus with subtomentose to cracked pileus surface, yellowish brown to greyish yellow in colour), differently featured stipe context (never turning greyish orange near base) and distinctively larger basidiospores (13.8–18.22–22 × 5.4–5.96–7 µm)^5^. The European *L. pseudoscabrum* (Kallenb.) Mikšíkis [= *L*. *carpini* (R. Schulz) Bresinsky & Manfr. Binder] is morphologically quite similar to *L*. *bothii* but differs by larger basidiomata [pileus 30–70 (–100) mm; stipe 60–130 × 6–14 mm], stipe that is entirely covered with brownish black dot-like squamules arranged in longitudinal rows, cutis pattern of stipitipellis and the occurrence under *Carpinus betulus* or *Corylus avellana*^9,11,12^.

The second species in this genus, *L. sinoaurantiacum* which was collected from East Khasi hills of Northeast India, is a very attractive mushroom for its beautiful scarlet to orange red basidiomata. Combination of macro- and micromorphological characters of Indian collections like scarlet to crimson red sticky pileus, yellow hymenophore with angular pores, scabrous stipe surface, comparatively long basidiospores, an ixohyphoepithelium nature of pileipellis confirm their identity as *L. sinoaurantiacum*^13,14^. Moreover, phylogenetic analysis of these collections (with LSU and *tef* 1-α) warrants this conspecificity of the Indian collections with its Chinese counterpart (voucher nos. Zang13486 and Li2770).

#### Xerocomoideae

**Figure 10:**
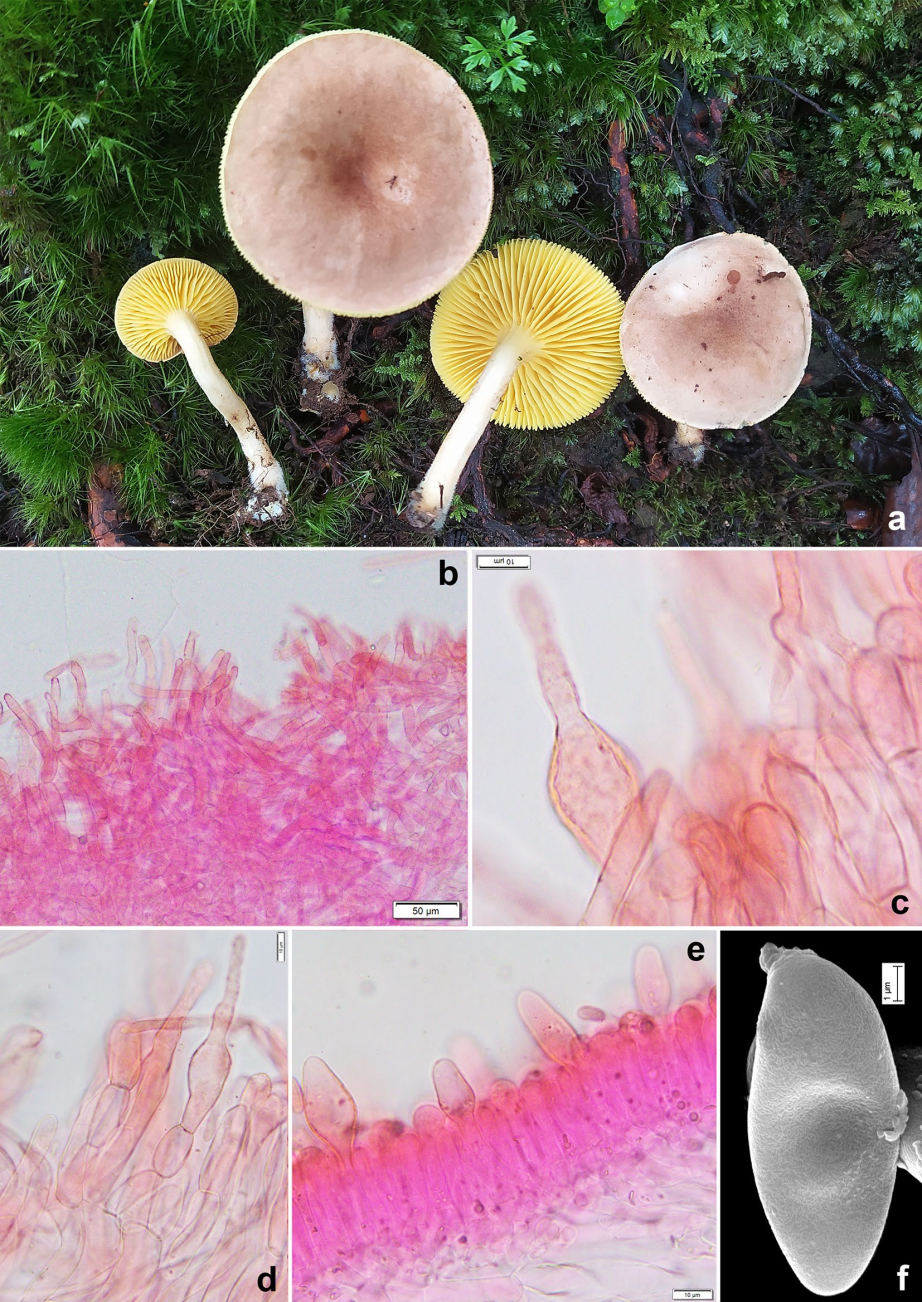
*Phylloporus himalayanus*. (KD 23-046, holotype). (**a**) Fresh basidiomata in the field. (**b**) Pileipellis. (**c**,**d**) Stipitipellis. (**e**) Hymenium layer with basidia and pleurocystidia. (**f**) Basidiospore under SEM. Scale bars (**b**) = 40 µm, (**c**–**e**) = 10 µm, (**f**) = 1 µm.

**Figure 11:**
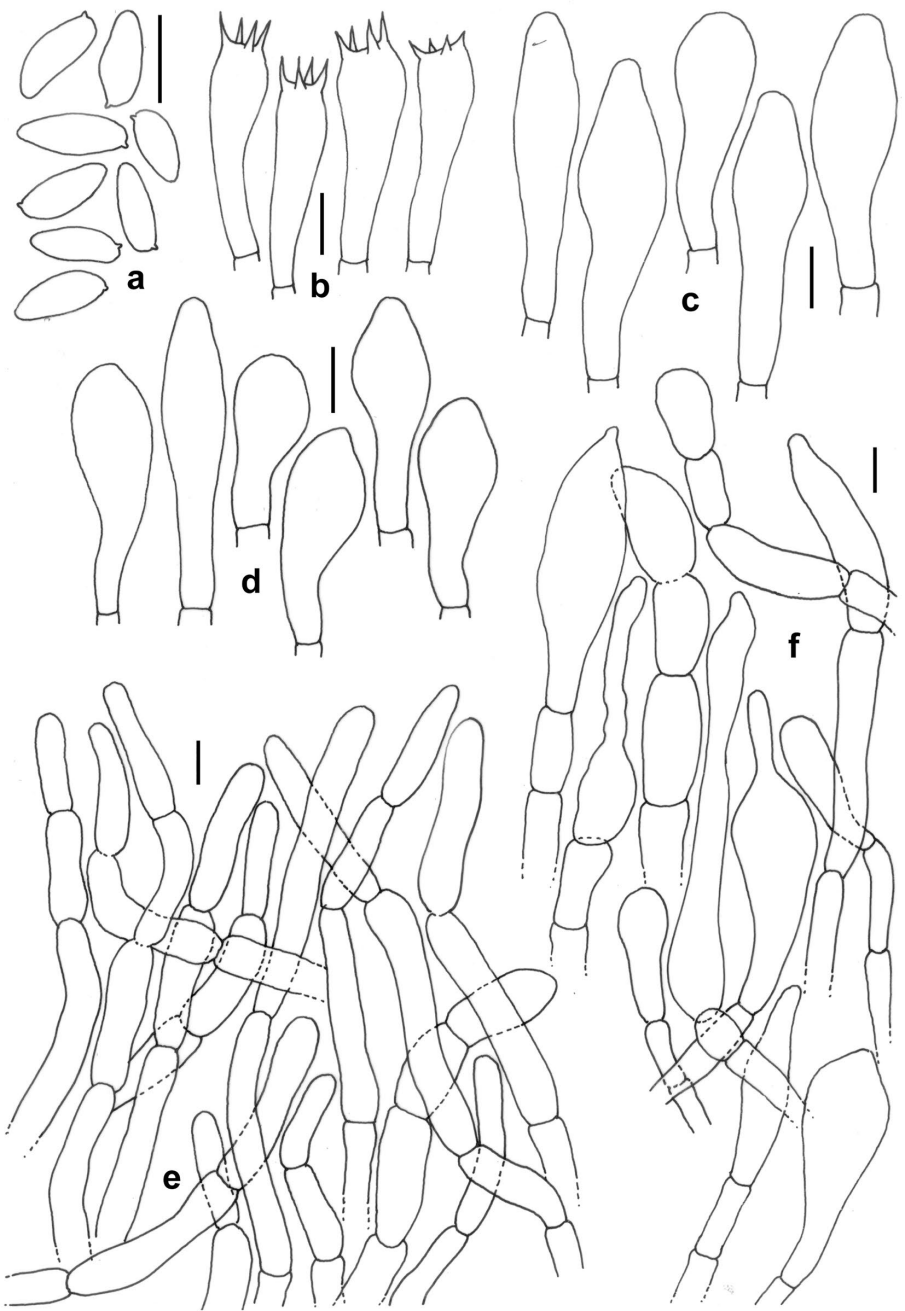
*Phylloporus himalayanus*. (KD 23-046, holotype). (**a**) Basidiospores. (**b**) Basidia. (**c**) Pleurocystidia. (**d**) Cheilocystidia. (**e**) Elements of pileipellis. (**f**) Elements of stipitipellis. Scale bars: (**a**–**f**) = 10 µm.

*Phylloporus himalayanus* K. Das, Sudeshna Datta & A. Ghosh sp. nov. *Mycobank*: MB 851129. *Holotype*: INDIA, Uttarakhand, Bageshwar district, 30° 04.270′ N 79° 55.229′ E, alt. 2870 m, on Dhakuri to Loharkhet trek close to Dhakuri-top, subalpine mixed forest under *Quercus* sp., 15 August 2023, *K. Das*, KD 23-046 (CAL 1955, holotype!) (Figs. 2, 10, 11).

*Etymology* Refers to the Himalayan Mountain range, where the type locality is situated.

*Diagnosis* Distinguished from the other known *Phylloporus* species by subdistant lamellae (8–10/10 mm), sub-bulbous and strigose stipe base, extremely varied hyphal terminal elements of stipitipellis and ITS, LSU and *tef* 1-α sequence data.

*Basidiomata* small to medium-sized, growing solitary to gregarious. *Pileus* 21–55 mm in diam., planoconvex with shallowly depressed centre, then applanate with depressed center; margin decurved when young, slightly uplifted; surface smooth to finely tomentose, brown (6E5–7) at centre, light brown (6D5) towards and along margin when young, gradually brownish orange (5C4–6) with pale orange to orange-white (5A2–3) along margin and darker centre at maturity, turning reddish brown (9E6–7) with KOH; context yellowish white (2A2) then brownish, turning greyish red (7B3) with KOH, greyish in FeSO_4_. *Hymenophore* lamellate, decurrent, subdistant (8–10/10 mm), intervenose and anastomosing, up to 8 mm in height, yellow to vivid yellow (3A6–8), becoming pastel green to turquoise green (25A4–5) very slowly; lamellulae in 5 series, attenuate, ventricose, concolorous with lamellae. *Stipe* central, 55–70 × 5–8 mm, subcylindric, dry, finely tomentose, with longitudinal striation on upper part, with sub-bulbous and strigose base, solid, pastel yellow to light yellow (3A4–5) towards apex, pale yellow (3A3) at middle, yellowish white (3A2) towards base with yellowish basal mycelium. *Context* in pileus yellowish white (2A2), becoming brownish, turning greyish red (7B3) with KOH; greyish with FeSO_4_; in stipe white to pale yellow (2A2–3), turning greyish red (7B3) with KOH, unchanging with FeSO_4_. *Basal mycelium* yellowish. *Annulus* absent. *Taste* not recorded. *Odour* indistinct. *Spore print* olive brown.

*Basidiospores* 7–9.4–11.5 × 3–3.9–4.5 μm, (n = 30, Q = 1.88–2.45–3), elliptical to oblong, olivaceous in 5% KOH, smooth under light microscope, but with bacillate ornamentation under SEM. *Basidia* 28–34 × 7.5–11.5 μm, subclavate to clavate, elongate, hyaline, 4-spored; sterigmata 2–4.5 × 0.5–1 μm. *Pleurocystidia* 36–49 × 9–14 μm, common, clavate with rounded or subfusoid apex, ventricose, emergent up to 24 μm. *Lamellae edge* fertile, composed of basidia and cystidia. *Cheilocystidia* 26–39 × 11–13 μm, common, clavate with rounded or subfusoid apex, emergent up to 18.5 μm. *Hymenium layer* up to 22 μm thick, hymenophoral trama composed of up to 8 μm wide cylindrical, smooth, hyaline, septate, parallel hyphae. *Pileipellis* 200–300 μm thick, an interwoven, compact trichoderm composed of erect to suberect, hyaline, septate, branched hyphae; terminal elements 22–69 × 6–11 μm, cylindrical, with rounded to obtuse apex. *Stipitipellis* up to 150 μm thick, a trichodermium, composed of erect to suberect, content dense to slightly granular in many or hyaline hyphae, forming; terminal elements 20–96 × 9–20 μm, inflated, clavate, ventricose to pyriform or bulbous with mucronate or lageniform apex or cylindrical with fusoid apex; subterminal elements of few hyphae inflated; caulobasidia not found. *Clamp connections* absent in all tissues.

*Additional specimen examined* INDIA, Uttarakhanad, Bageshwar district, on way between Dhakuri and Loharkhet, 30° 04.084′ N 79° 55.195′ E, alt. 2849 m, subalpine mixed forest under *Quercus* sp., 15 August 2023, *K. Das*, KD 23-047 (CAL 1956).

**Figure 12:**
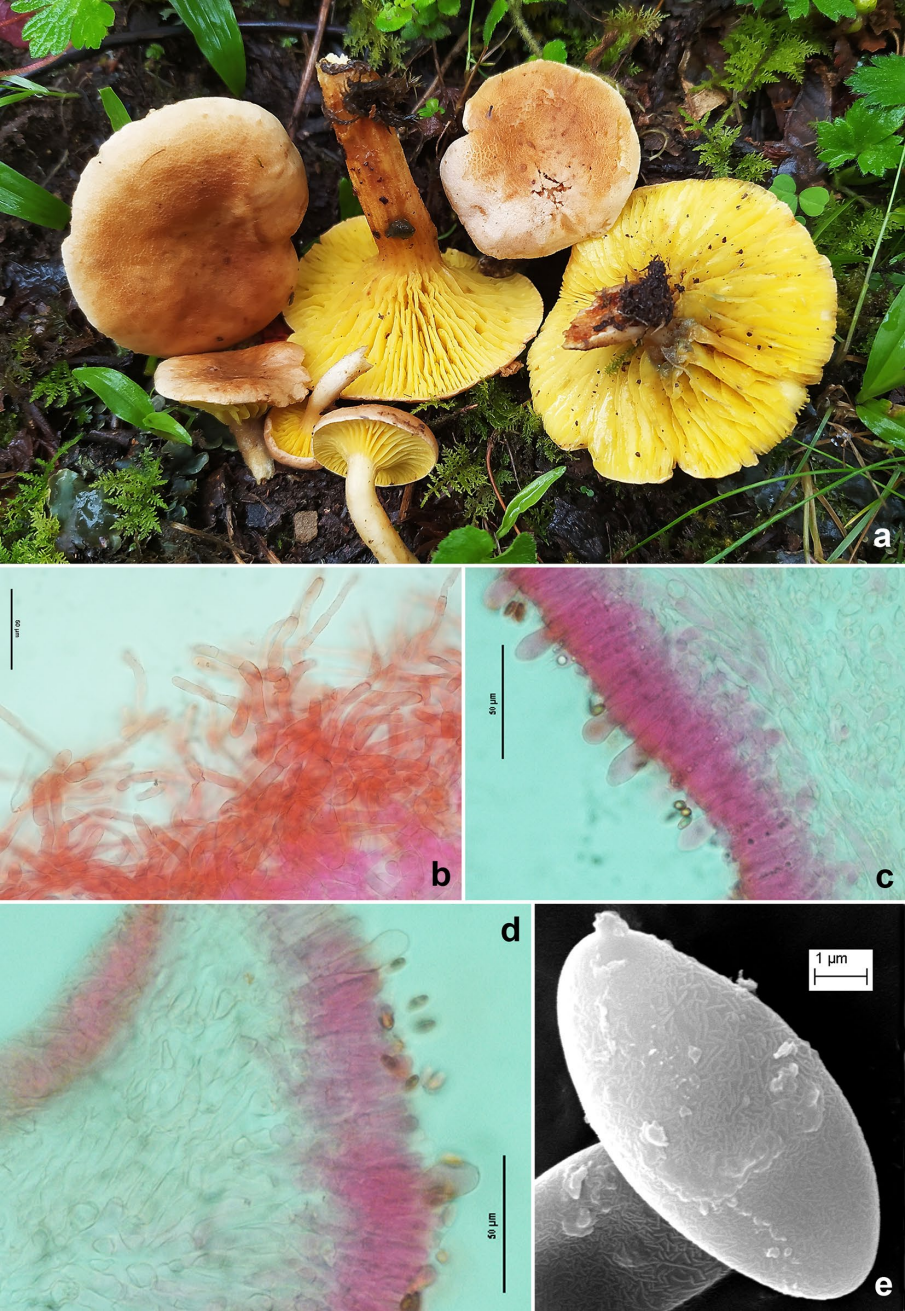
*Phylloporus smithii*. (KD 23-012, holotype). (**a**) Fresh basidiomata in the field. (**b**) Pileipellis. (**c**) Pleurocystidia. (**d**) Cheilocystidia. (**e**) Basidiospores under SEM. Scale bars: (**b**–**d**) = 50 µm, (**e**) = 1 µm.

**Figure 13:**
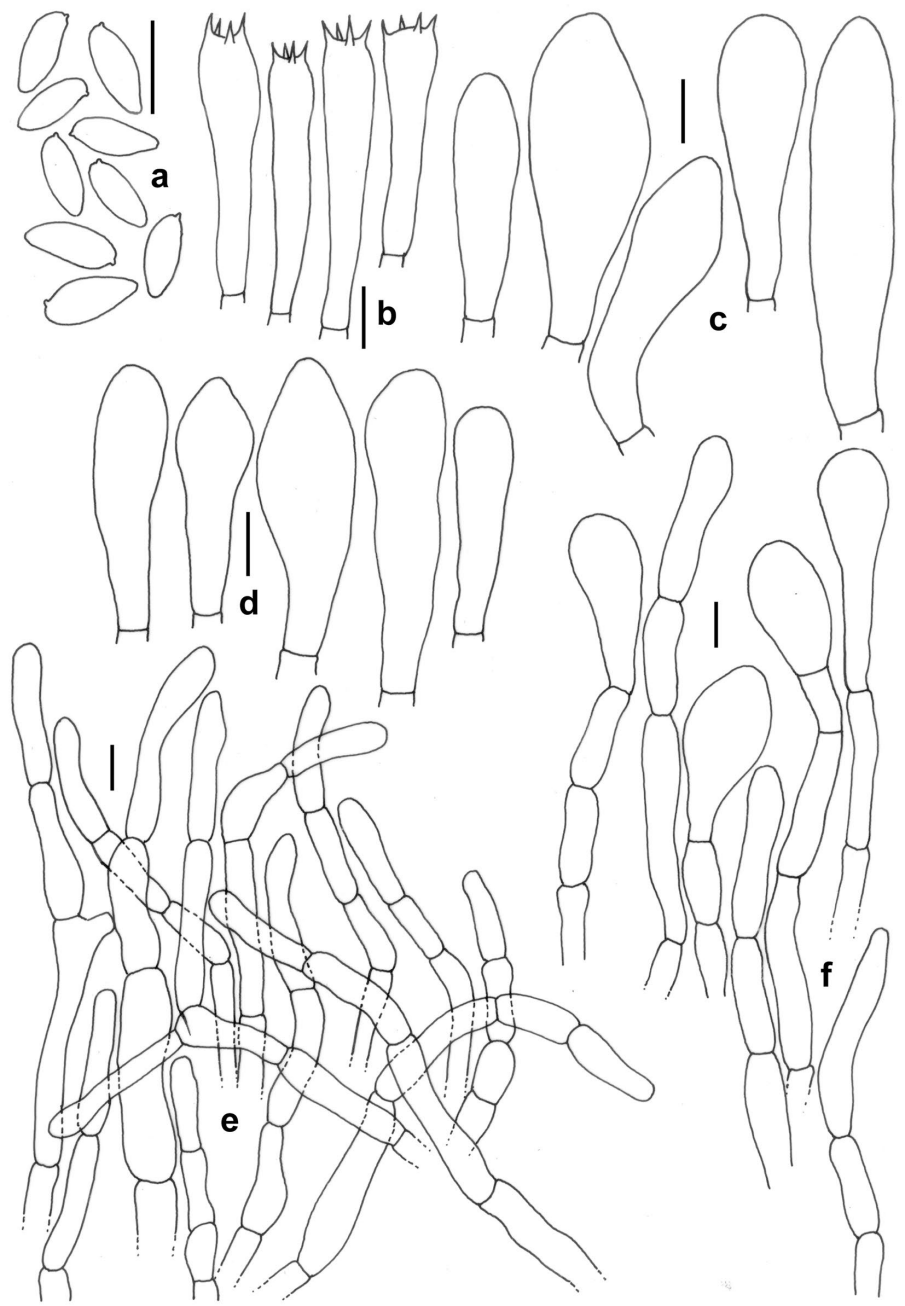
*Phylloporus smithii*. (KD 23-012, holotype). (**a**) Basidiospores. (**b**) Basidia. (**c**) Pleurocystidia. (**d**) Cheilocystidia. (**e**) Elements of pileipellis. (**f**) Elements of stipitipellis. Scale bars: (**a**–**f**) = 10 µm.

*Phylloporus smithii* K. Das, Sudeshna Datta, U. Singh & A. Ghosh sp. nov. *Mycobank*: MB 851130. *Holotype*: INDIA, Uttarakhand, Rudraprayag district, Baniyakund, 30° 10.146′ N 078° 52.107′ E, alt. 2563 m, temperate mixed forest under *Quercus* sp., 4 August 2023, *K. Das*, KD 23-012 (CAL 1957, holotype!) (Figs. 2, 12, 13).

*Etymology* Commemorating Alexander H. Smith for his significant contribution to the systematics of Boletaceae.

*Diagnosis* Distinguished from the other known *Phylloporus* species by minutely cracked pileus surface, rather crowded lamellae (18–20/10 mm), stipe that is gradually tapering towards base and ITS, LSU and *tef* 1-α sequence data.

*Basidiomata* small to medium-sized, solitary to gregarious. *Pileus* 15–54 mm in diam., convex to planoconvex with shallowly depressed to flat centre, then applanate, finally somewhat funnel-shaped with depressed centre, margin decurved; surface somewhat velvety and minutely cracked with maturity, brown (6D6) to light yellow (4A4) at or near centre, paler towards margin; violet-brown (10E7) with KOH, greenish with FeSO4; context off white, unchanging in color when injured. *Hymenophore* lamellate; lamellae decurrent, close to rather crowded (18–20/10 mm), intervenose and anastomosing, up to 5 mm in height, yellow (2A6), slowing becoming blue (25D4) when bruised; lamellulae in 4 series, attenuate, ventricose, concolorous with lamellae. *Stipe* central, 18–50 × 3–10 mm, subcylindric with distinctively tapering base, solid; pale yellow (3A3) when young, gradually brownish from middle to base; surface dry, tomentose upper part sometimes ribbed to striate by the decurrent lines of the lamellae. *Context* in pileus off-white with pale yellow centre, unchanging on exposure. *Basal mycelium* whitish. *Annulus* absent. *Taste* not recorded. *Odour* indistinct. *Spore print* olive brown.

*Basidiospores* 8.5–9.8–11.5 × 3–4.1–5 μm, (n = 30, Q = 2–2.4–3), elliptical to oblong, olivaceous (1C2) in 5% KOH, smooth under light microscope, but with bacillate ornamentation under SEM. *Basidia* 33–44 × 6–10 μm, subclavate to clavate, elongate, hyaline, 4-spored; sterigmata 1–4 × 0.5–1 μm. *Pleurocystidia* 24–62 × 8–19 μm, common, subclavate to broadly clavate with rounded or subfusoid apex, rarely fusiform, septate, emergent up to 20 μm. *Lamellae edge* fertile, composed of basidia and cystidia. *Cheilocystidia* 34–50 × 9–14 μm, common, clavate with rounded or subfusoid apex, rarely subventricose, emergent up to 20 μm. *Hymenium layer* up to 37 μm thick; hymenophoral trama composed of up to 7 μm wide cylindrical, smooth, hyaline, septate, parallel hyphae. *Pileipellis* 150–200 μm thick, a trichodermium, composed of erect to suberect hyaline, septate, rarely branched hyphae; terminal elements 20–47 × 5–9 μm, cylindrical, with rounded to subfusoid apex. *Stipitipellis* up to 150 μm thick, a trichodermium, composed of erect to suberect, hyaline hyphae; terminal elements 29–53 × 11–17 μm, cylindrical or clavate, bulbous to pyriform or cylindrical with fusoid apex, subterminal elements occasionally inflated; *caulobasidia* similar to tube basidia. *Clamp connections* absent in all tissues.

*Additional specimen examined* INDIA, Uttarakhand, Chamoli district, Didna top, 30° 09.922′ N 79° 38.042′ E, alt. 2536 m, temperate mixed forest under *Quercus* sp., 8 August 2023, *K. Das*, KD 23-022 (CAL 1958).

*Notes* Basidiomata with strong decurrent intervenose to anastomosing lamellae (instead of poroid hymenophore) and bacillate spores place the two proposed species under *Phylloporus* Quél.^15,16^ among boletoid fungi. It is realized that due to phenotypic plasticity in this genus, morphology-based species identification is quite impossible. Concordance of multigene genealogy along with morphology is the only solution to separate these species having overlapping morphological features. Present species, *P. smithii* is distinctively characterised by the pileus surface being minutely cracked, rather crowded lamellae (18–20/10 mm) and stipe that is gradually tapering from apex to base whereas, *P. himalayanus* is significantly featured by subdistant lamellae (8–10/10 mm), typically sub-bulbous strigose stipe base, diversified terminal elements of stipitipellis hyphae and absence of caulobasidia. These two species can be separated in the field itself.

*Phylloporus himalayanus* looks like *P. yunnanensis* N.K. Zeng, Zhu L. Yang & L.P. Tang (originally reported from China) and *P. subrubeolus* Chuankid, K.D. Hyde & Raspé (originally reported from Thailand). However, both *P. yunnanensis* and *P. subrubeolus* are distinguished from *P. himalayanus* by the absence of strigose sub-bulbous stipe base and microscopically, they lack terminal elements with a mucronate, lageniform to appendiculate apex in the hyphae of stipitipellis^15,17^. Similarly, *P. smithii* appears quite close to *P. imbricatus* N.K. Zeng, Zhu L. Yang & L. P. Tang, another Asian species originally reported from China. However, the later can be separated by the distinctively larger (50–100 × 3–15 mm) stipe, subdistant lamellae and microscopically, by larger basidiospores (10–13 × 4–5 μm), fertile stipitipellis and fusiform pleurocystidia^15^. Moreover, our multigene molecular phylogenetic estimation clearly separates these two Indian species among themselves and from the other known species of *Phylloporus* as shown in Fig. 2.

**Figure 14:**
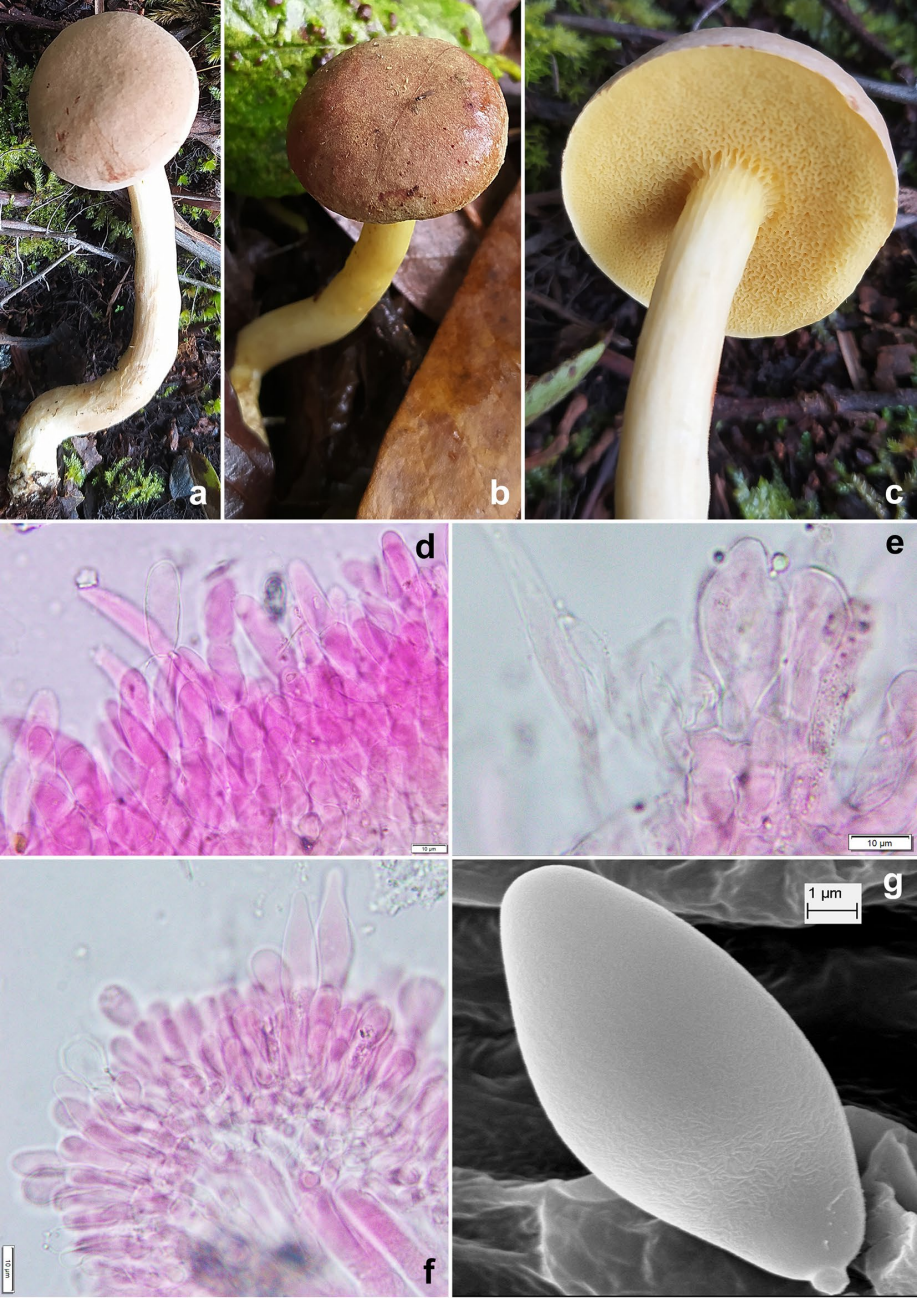
*Xerocomus rugosellus* (KD 23-019). (**a**–**c**) Fresh basidiomata. (**d**) Pileipellis. (**e**) Caulocystidia. (**f**) Cheilocystidia. (**g**) Basidiospore under SEM. Scale bars: (**d**–**f**) = 10 µm, (**g**) = 1 µm.

**Figure 15:**
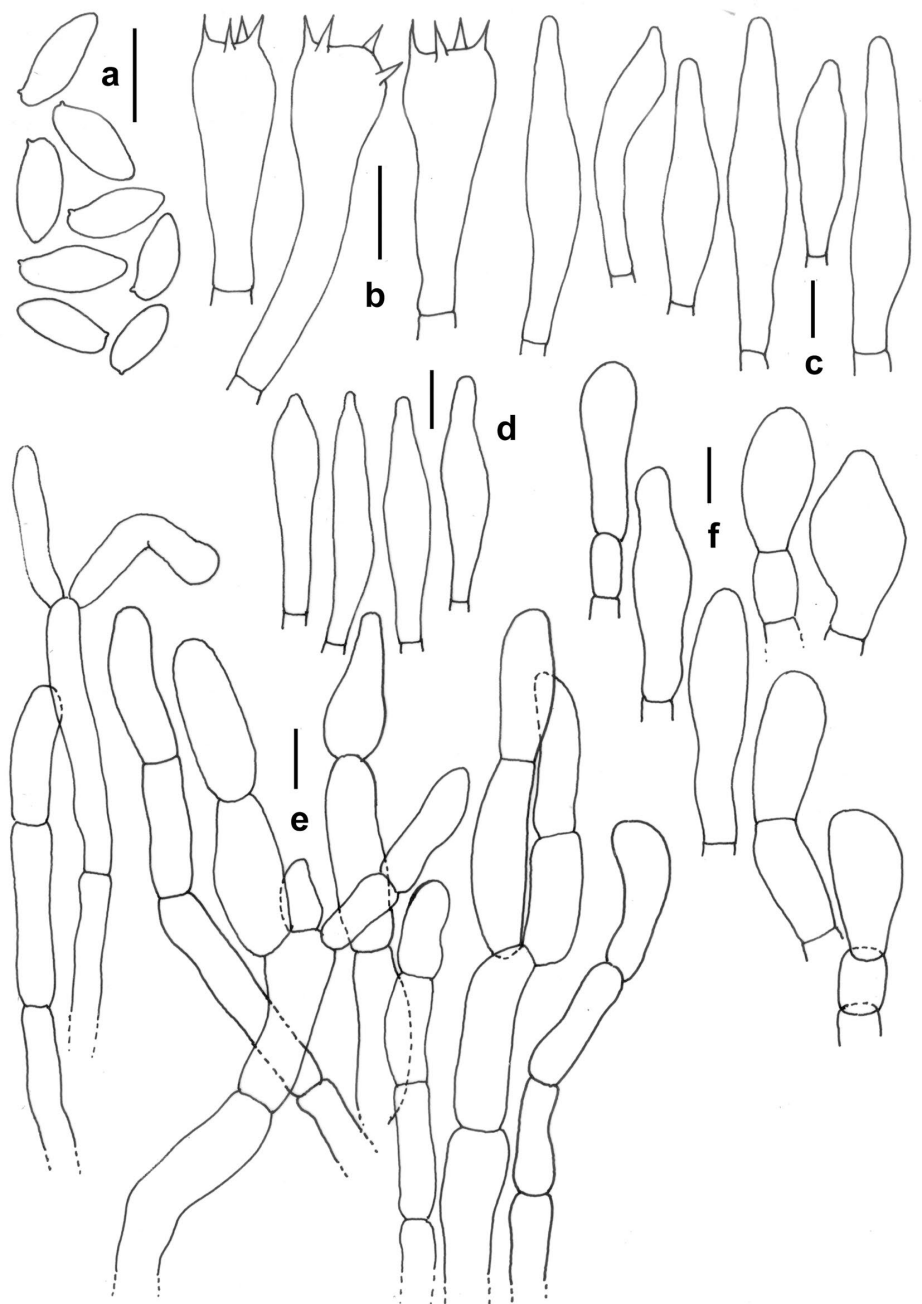
*Xerocomus rugosellus* (KD 23-019). (**a**) Basidiospores. (**b**) Basidia. (**c**) Pleurocystidia. (**d**) Cheilocystidia. (**e**) Elements of pileipellis. (**f**) Caulocystidia. Scale bars: (**a**–**f**) = 10 µm.

*Xerocomus rugosellus* (W.F. Chiu) F.L. Tai, *Syll. fung. sinicorum*: 815 (1979) (Figs. 3, 14, 15).

*Basidiomata* small to medium-sized. *Pileus* 40–50 mm in diam., convex when young, becoming planoconvex with maturity; surface rugose to subtomentose, non-viscid, greenish yellow (4C5) to pompeian yellow (5C6) or paler; margin entire, with a very narrow sterile flap of tissue; turning brown (7E8) with KOH. *Pore surface* yellow (3A6–7), initially unchanging when bruised, later becoming blue green; pores ellipsoid to elongate or bacillate, often compound, 1–2/mm. *Tubes* adnate to adnexed, 4–4.3 mm long, light yellow (2A5), unchanging when bruised or exposed. *Stipe* 85–95 × 8–10 mm, more or less cylindrical, gradually tapering towards the base; surface reticulate at apex, then longitudinally ridged or striated towards mid, yellowish white at the apex, orange-white (6A2) towards the middle and lower half. *Context* in pileus up to 12 mm thick, pale to pastel yellow (2A3–4), turning light orange (5A4) in KOH and slightly greenish with FeSO_4_; context in stipe solid to pithy, light yellow (2A5) on the upper 1/3rd of the stipe length or paler, lower 2/3rd brownish, pith brown (6E5–6). *Basal mycelium* white. *Odour* mild. *Spore print* not obtained.

*Basidiospores* 8.5–10.5–13 × 3.5–4.7–6 µm, (n = 30, Q = 1.98–2.25–2.89), ellipsoid to fusoid and inequilateral in side view, hyaline, smooth under light microscope but under SEM spore surface bacillate. *Basidia* 27–40 × 9–11 µm, clavate, 4-spored; sterigmata 2–5 × 0.5–1 µm. *Pleurocystidia* 36–60 × 8–11 µm, ventricose or fusoid, thin-walled, with finely granular content, emergent up to 37 µm. Tube edge fertile. *Cheilocystidia* 34–45 × 7–8.5 µm, less frequent, ventricose or fusoid, thin-walled, emergent up to 33 µm. *Hymenophoral trama* composed of thin-walled, septate, parallel; hyphae up to 10 µm wide, branched, septate. *Pileipellis* up to 150 µm thick, a trichodermium, composed of erect to suberect, cylindrical, regularly septate hyphae, sometimes branched, thin-walled, few with dense content, olive with 5% KOH; terminal elements 15–40 × 5–11 µm, cylindrical, sometimes clavate, or ventricose to fusiform. *Stipitipellis* up to 250 µm thick, fertile, composed of thin-walled, branched, septate, parallelly arranged hyphae and few tufts of basidia, basidioles, and caulocystidia (caulohymenium); *caulocystidia* 27–42 × 10–17 µm, subclavate to clavate, ventricose, pyriform to bulbous; *caulobasidia* 33–40 × 9–10 µm, 4-spored. *Clamp connections* absent in all tissues.

*Specimens examined* INDIA, Uttarakhand, Chamoli district, Didna top, 30° 09.922′ N 79° 38.042′ E, alt. 2536 m, temperate mixed forests under *Quercus* sp., 8 August 2023, *K. Das*, KD 23-019 (CAL 1964); *ibid.*, Bageshwar district, on way between Dhakuri and Khati, 30° 04.934′ N 79° 55.080′ E, alt. 2545 m, temperate mixed forests under *Quercus* sp., 14 August, 2023, *K. Das*, KD 23-055 (CAL 1965).

*Notes* Present species is the first report for the Indian mycobiota. In the field, *Xerocomus rugosellus* is characterized by the rugose pileus surface (when young), the slowly bluing pore surface and context (on bruising), comparatively tall and slender stipe. Our Indian collections are in conformation with the holotype (Chinese material) except the hymenial cystidia and basidiospores which are comparatively small in present collections^16,18^. Few Asian (Indian) species that share morphological and molecular affinities with the present species are *Xerocomus doodhcha* K. Das, D. Chakr., A. Baghela, S.K. Singh & Dentinger, *X. longistipitatus* K. Das, A. Parihar, D. Chakr. & A. Baghela, *X. uttarakhandae* K. Das, Sudeshna Datta, and A. Ghosh and *X. reticulostipitatus* Hembrom, D. Chakr., A. Parihar & K. Das. However, *X. doodhcha* is distinct by a typical “milk–tea” colour of pileus, comparatively shorter stipe (50–68 × 4–10 mm) and angular pores^19^. *Xerocomus longistipitatus* has robust basidiomata with brown pileus and exceptionally long stipe (70–185 × 10–24 mm), pore surface that turns greenish grey to dull green slowly on bruising, angular to irregular pores^20^, whereas *X. reticulostipitatus* shows very prominent and typical brownish red to reddish brown reticulation on stipe and distinctively larger basidiospores (10.3–12.2–15.6 × 3.7–4.4–5.3 µm)^21^. *Xerocomus uttarakhandae* is segregated (from *X. rugosellus*) by possessing typically cracked to areolate greyish orange to greyish brown pileus surface exposing inner reddish context^5^.

#### Boletoideae

**Figure 16:**
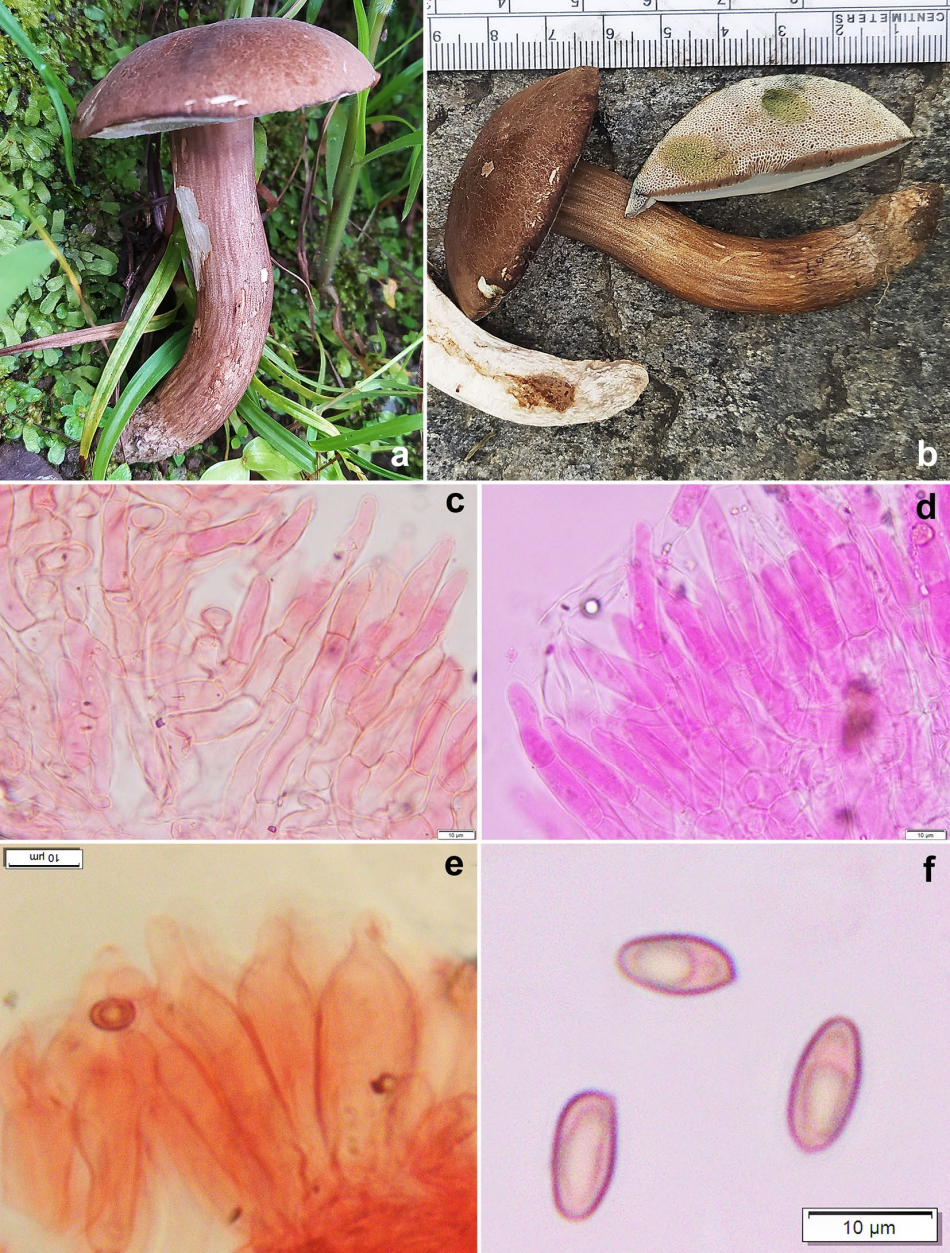
*Porphyrellus uttarakhandae*. (KD 23-028, holotype). (**a**,**b**) Fresh basidiomata. (**c**,**d**) Pileipellis. (**e**) Caulocystidia. (**f**) Basidiospores. Scale bars: (**c**–**f**) = 10 µm.

**Figure 17:**
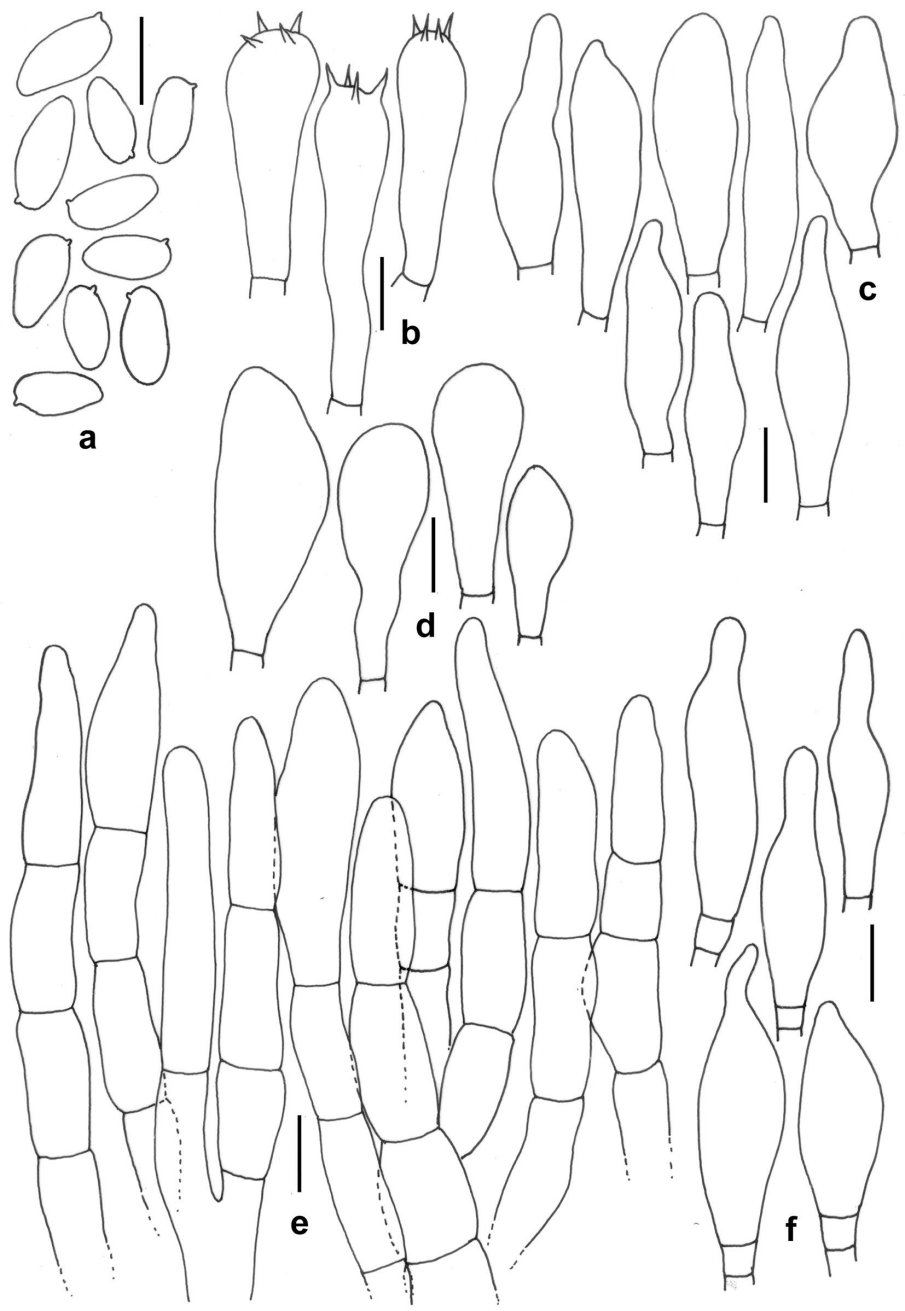
*Porphyrellus uttarakhandae*. (KD 23-028, holotype). (**a**) Basidiospores. (**b**) Basidia. (**c**) Pleurocystidia. (**d**) Cheilocystidia. (**e**) Elements of pileipellis. (**f**) Caulocystidia. Scale bars: (**a**–**f**) = 10 µm.

*Porphyrellus uttarakhandae* K. Das, Sudeshna Datta & A. Ghosh sp. nov. *Mycobank*: MB 851131. *Holotype*: INDIA, Uttarakhanad, Chamoli district, Lohajung, 30° 27.811′ N 79° 16.178′ E, alt. 2283 m, temperate mixed forests under *Quercus* sp., 10 August 2023, *K. Das*, KD 23-028 (CAL 1959, holotype!) (Figs. 4, 16, 17).

*Etymology* referring to the Himalayan state of Uttarakhand, where the type locality is situated.

*Diagnosis* Distinct from closely allied species i.e., *P. orientifumosipes* by shorter tubes, absence of a bluish ring like zone on stipe apex, larger basidiospores, shorter hymenial cystidia and LSU, *rpb2*, and *tef* 1-α sequence data.

*Basidiomata* small to medium-sized. *Pileus* 45–65 mm diam., sub-hemispherical to convex or at the most planoconvex, yellowish brown (5D–E8) to light brown (7D4–5) to reddish brown or umber with slightly darker in the center; surface dry, minutely cracked into small squamules on a whitish background; margin decurved with a flap of tissue of 0.8 mm wide by diam. *Hymenophore* adnexed to sinuate when young, depressed around apex of stipe when mature; pore surface whitish to pinkish to brownish pink, turning asymmetrically greyish turquoise (24D5–6) or greenish blue when bruised; pores subangular to roundish, 1–2/mm; tubes up to 7 mm long, concolorous to pore surface, turning faint greenish blue when exposed. *Stipe* cylindrical, 50–75 × 8–13 mm, concolorous to pileus surface; surface minutely cracked. *Context* in pileus, chalky to greyish white, asymmetrically greenish blue or paler when exposed; in stipe chalky up to mid, greyish white towards base, asymmetrically greenish blue or paler when exposed. *Basal mycelium* whitish to greyish white, unchanging when bruised. *Taste and odour* mild. *Spore print* orange-red to brownish red.

*Basidiospores* 8.7–11–13.7 × 5–5.4–6.2 µm (n = 30, Q = 1.64–2.01–2.45), broadly subfusiform to ellipsoid, inequilateral in sideview, smooth under light microscope. *Basidia* 36–45 × 9–14 µm, clavate, elongate, 4-spored; sterigmata 3–5 × 1–2 µm. *Pleurocystidia* 34–46 × 7–12 µm, fusiform, clavate to subventricose with rounded apex or appendiculate apex, thin walled, hyaline; emergent up to 26 µm. Tube edge fertile, composed of basidia, basidiole and cystidia. *Cheilocystidia* 24–40 × 9–17 µm, broadly clavate to pyriform, thin-walled, hyaline; emergent up to 22 µm. *Hymenophoral trama* divergent, composed of compactly arranged, septate, thin-walled hyphae, 5–7 µm wide. *Pileipellis* up to 150 µm thick, a trichodermium to palisadoderm, composed of compactly arranged, branched, septate, erect, thin-walled hyphae with chains of slightly inflated elements; terminal elements 18–47 × 6–12 µm, cylindrical to subcylindrical, clavate, subfusiform with rounded or tapering apex, rarely bulbous. *Stipitipellis* up to 50 µm thick, composed of irregularly arranged, branched, septate, erect, thin-walled hyphae, with infrequent tuft of basidia and cystidia; *caulocystidia* 27–53 × 8–13 µm, ventricose to subfusiform with rounded, subcapitate to appendiculate apex, thin-walled, hyaline; *caulobasidia* 37–44 × 13–16 µm, broadly clavate, 4-spored. *Clamp connections* absent in all tissues.

*Additional specimen examined* INDIA, Uttarakhand, Chamoli district, Kuling, 30° 27.811′ N 79° 16.178′ E, alt. 2296 m, temperate mixed forests under *Quercus* sp., 10 August 2023, *K. Das*, KD 23-056 (CAL 1960).

*Notes* Possession of umber coloured basidiomata, whitish or greyish context without significant discoloration or becoming asymmetrically greenish blue, white to pinkish pore surface that changes asymmetrically greenish blue, palisadoderm pattern of pileipellis, and smooth basidiospores place the present species under *Porphyrellus* E.-J. Gilbert^1,3^.

Two Asian species namely, *Por. orientifumosipes* and *Por. pseudocyaneotinctus* look quite similar to *Por. uttarakhandae* in the field. However, *Por. orientifumosipes* differs mostly from the present species by longer tubes (up to 20 mm), presence of ring-like bluish zone at stipe apex, smaller basidiospores (9.5–10.5 × 4.5–5.5 µm) and much longer hymenial cystidia (58–74 × 15–19 µm) whereas, *Por. pseudocyaneotinctus* shows distinctively more robust basidiomata (pileus 46–99 mm in diameter, stipe 48–123 × 9–19 mm), larger hymenial cystidia (pleurocystidia 36.8–85 × 8.5–13 µm, cheilocystidia 38.2–60.5 × 10.8–17.9 µm), differently-shaped cheilocystidia (lageniform), differently-shaped terminal elements of pileipellis hyphae and a sterile stipitipellis^18,38^.

**Figure 18:**
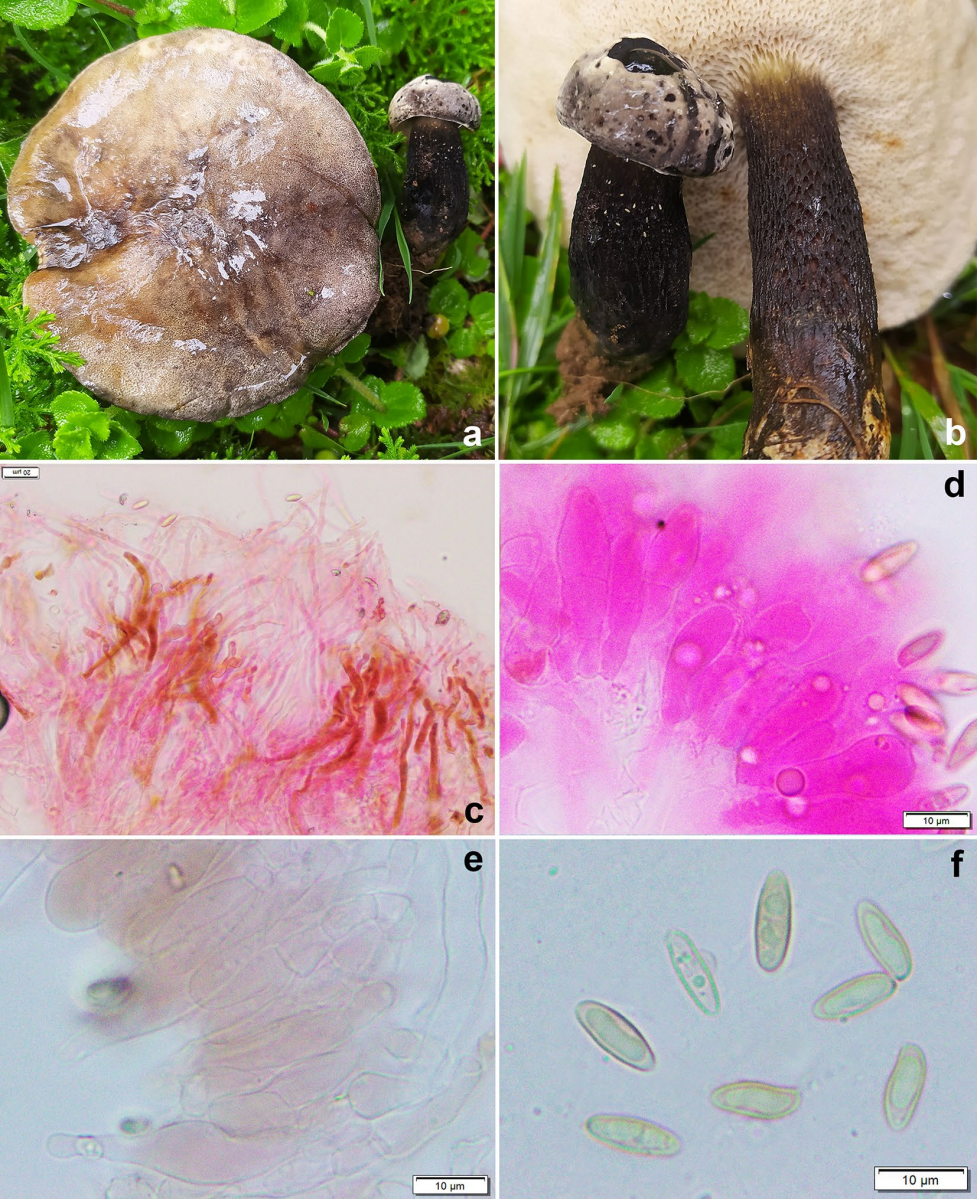
*Retiboletus pseudoater*. (KD 23-040, holotype): (**a**,**b**) Fresh basidiomata (**c**) Pileipellis (**d**) Cheilocystidia (**e**) Stipitipellis (**f**) Basidiospores. Scale bars (**c**) = 20 µm, (**d**–**f**) = 10 µm.

**Figure 19:**
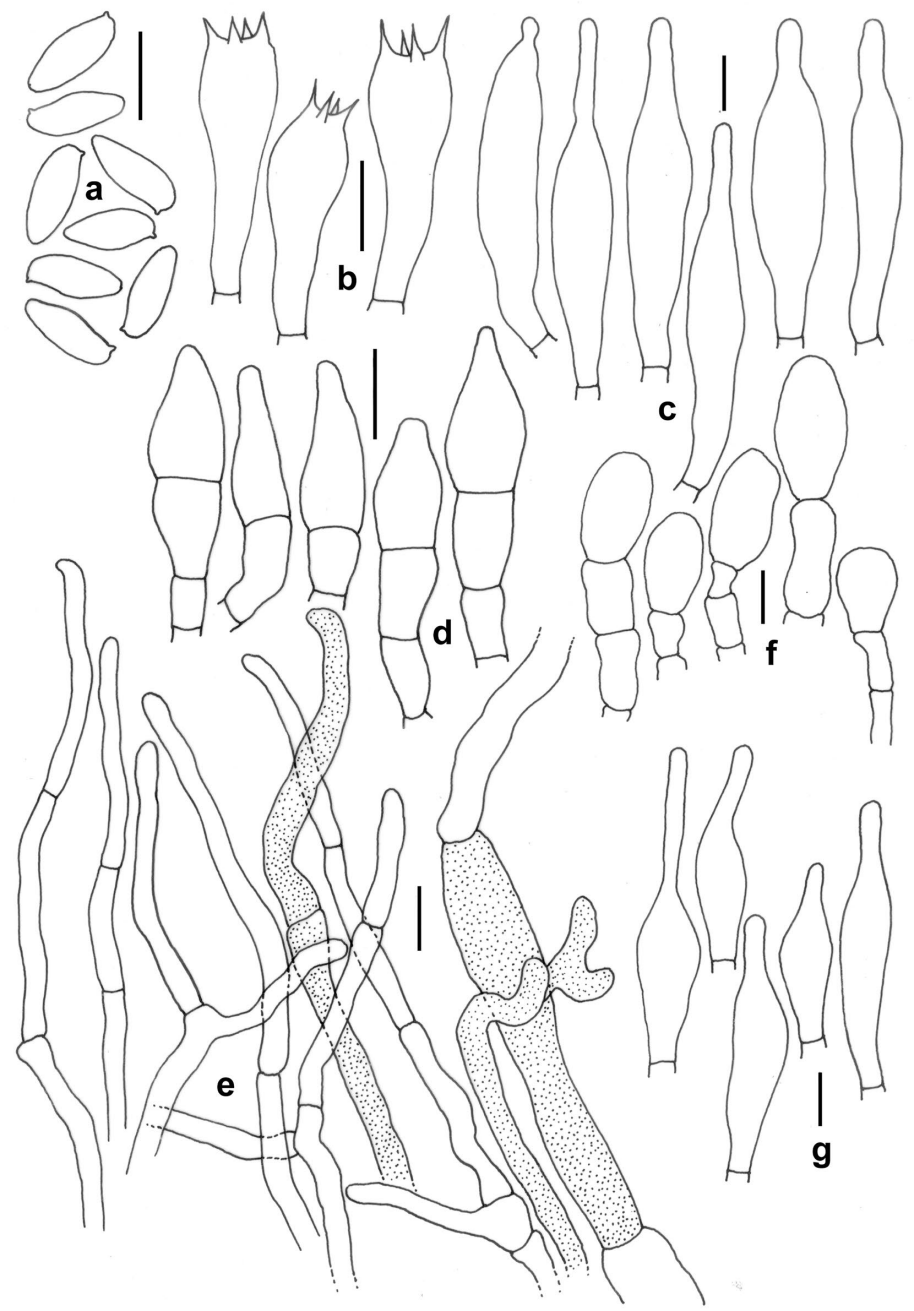
*Retiboletus pseudoater*. (KD 23-040, holotype). (**a**) Basidiospores. (**b**) Basidia. (**c**) Pleurocystidia. (**d**) Cheilocystidioid elements. (**e**) Elements of pileipellis. (**f**) Hyphal elements of stipitipellis showing terminal and subterminal elements. (**g**) Caulocystidia. Scale bars: (**a**–**f**) = 10 µm.

*Retiboletus pseudoater* K. Das, A. Ghosh, Sudeshna Datta & Vizzini sp. nov. *Mycobank*: MB 851132. *Holotype*: INDIA, Uttarakhanad, Bageshwar district, on way between Dhakuri to Khati, 30° 04.934′ N 79° 55.080′ E, alt. 2545 m, temperate mixed forest under *Quercus* sp., 14 August 2023, *K. Das*, KD 23-040 (CAL 1961, holotype!) (Figs. 5, 18, 19).

*Etymology* Referring to the morphological similarity of the species with *Retiboletus ater* another Asian species.

*Diagnosis* Distinguished from the closely allied *R. ater* by larger (22–90 mm in diameter) pale orange to greyish orange or brownish orange pileus, presence of cheilocystidioid elements at lamellar edges and different ITS, LSU, and *tef* 1-α sequence data.

*Basidiomata* small to medium-sized. *Pileus* 22–90 mm in diam., hemispherical to convex, becoming planoconvex at maturity; surface dry, velvety, pale orange (5A3) to greyish orange (5B4) with patches of grey to greyish brown (5F1–3) or completely black when young, becoming greyish orange (5B3), brownish orange (5C4), light brown to cinnamon brown (6D4–5) in combination brownish grey to negro (6F2–3) with distinctive blackish dots or darker, unchanging with KOH; margin with a flap of tissue of 1 mm wide. *Hymenophore* adnate to adnexed; pore surface yellowish white to pale yellow (2–3A2–3), becoming nougat (5D3) initially, then slowly becoming to drab grey (5E3); pores angular, 2/mm. *Tubes* up to 10 mm long, concolorous with pore surface. *Stipe* 47–65 × 12–14 mm, elongate, subclavate, solid towards apex stuffed below; surface dry, grey to black prominent and coarse reticulation till more than middle of the stipe (but never extended up to base) on the background of absinthe yellow to olive yellow (3C5–6) or darker; base smooth, pale orange (5A3). *Context* in pileus, 12 mm thick at the centre, yellowish white (3–4A2), turning to reddish or pinkish white (7A2) with KOH, greenish grey (28B2) with FeSO_4_, in stipe yellowish white (3A2) at apex, gradually pastel yellow to light yellow (3A4–5) or deep yellow towards mid to base, turning reddish brown to mahogany (8E5–7) with KOH, greenish grey (28B2) with FeSO_4_. *Basal mycelium* white (2A1). *Taste* and *odour* indistinct. *Spore print* not obtained.

*Basidiospores* 10.2–11.29–12.9 × 3.4–4.2–4.9 μm (n = 30, Q = 2.36–2.65–3.1), subfusiform, elliptical to oblong, inequilateral in side view, elliptical to oblong, olivaceous (1C2) in 5% KOH, smooth. *Basidia* 25–30 × 8–10 μm, clavate, hyaline, 4-spored; sterigmata 2–4.5 × 0.5–1 μm. *Pleurocystidia* 57–70 × 11–13 µm, ventricose with subcapitate, capitate or appendiculate apex, thin-walled, with brown pigment, emergent up to 31 µm. Lamellae edge fertile, composed of basidia and cystidioid elments. *Cheilocystidioid* elements 30–38 × 7–10 μm, subfusiform to ventricose, 2–3 septate, terminal elements fusoid. *Hymenophoral trama* parallel; hyphae thin-walled, septate, cylindrical, 6–9 μm wide, hyaline to yellowish in KOH, yellowish to brownish yellow in Melzer’s reagent. *Pileipellis* 140–160 µm thick, a trichodermium, composed of erect to suberect, cylindrical, regularly septate branched thin-walled hyphae, mostly with brown intracellular pigmentation; terminal elements 15–40 × 5–11 µm, cylindrical. *Stipitipellis* up to 80 μm thick, mainly hymeniform with clusters of cystidia and basidia; terminal elements of 10–20 × 8–14 μm, subclavate to broadly clavate, or bulbous, some with brown pigmentation in KOH; subterminal elements often inflated; *caulocystidia* 34–60 × 9–12 μm, fusiform with lageniform, appendiculate or mucronate apex, ventricose to obclavate; *caulobasidia* 25–35 × 8–10 μm, clavate, 4-spored. *Clamp connections* absent in all tissues.

*Additional specimens examined* INDIA, Uttarakhand, Bageshwar district, Dhakuri to Loharkhet trek, 30° 04.270′ N 79° 55.229′ E, alt. 2870 m, subalpine mixed forest under *Quercus* sp., 15 August 2023, *K. Das*, KD 23-048 (CAL 1962); *ibid.*, Dhakuri to Dhur trek, 30° 05.009′ N 79° 53.882′ E, alt. 2538 m, temperate mixed forest under *Quercus* sp., 16 August 2023, *K. Das*, KD 23-051 (CAL 1963).

*Notes* Distinctive features of basidiomata like coarsely reticulate stipe surface and vivid yellow stipe context place this species under the genus *Retiboletus*^22^. Moreover, presence of pale orange to greyish brown to black pileus surface places the present species in recently established subgenus: *R.* subg. *Nigroretiboletorum* Yan C. Li & Zhu L. Yang^23^. This species can easily be distinguished from other species of *Retiboletus* by combination of features like pale orange, brownish orange to greyish orange, black dotted pileus, yellowish white to pale yellow pore surface, greyish black to black coarse reticulation on stipe surface, microscopically, by presence of typically 2–3 septate cheilocystidioid elements, and occurrence under *Quercus* species in subalpine Himalaya. In the field, this species resembles *R. ater* Yan C. Li & T. Bau (originally reported from China) however, the latter can be distinguished from the earlier by a smaller pileus (30–50 mm in diam.), comparatively smaller basidiospores “[60/3/2] (7)8–10.5(11) × 3–4.5(5) μm, absence of orange with black-dotted pileus surface, 2–3 septate cheilocystidioid elements, differently shaped (fusiform with lageniform, appendiculate or mucronate apex, ventricose to obclavate) caulocystidia and the presence of differently-shaped terminal elements of pileipellis (“narrowly clavate to subcylindrical or subfusiform, sometimes narrowly mucronate, rostrate”^24^). Few other species namely, *R. fuscus* (Hongo) N.K. Zeng & Zhu L. Yang, *R. nigrogriseus* N.K. Zeng, S. Jiang & Zhi Q. Liang and *R. pseudogriseus* N.K. Zeng & Zhu L. Yang (all originally reported from China) also share some features with *R. pseudoater*. However, all three species are characterized by overall reticulate stipe and the absence of orange tinges on pileus surface^25,26^. Another species reported from China, viz. *R. kauffmanii* (Lohwag) N.K. Zeng & Zhu L. Yang is easily separated in the field from our present species by grey-brown to brown pileus, yellow pore surface (2A5–6) and yellow reticulation on stipe surface^27^. Another Chinese species, *R. sinogriseus* Yan C. Li & T. Bau is also partly similar to the present novel species, however the former has stipe with pale yellow apex, blackish-yellow towards the base and thinner pileipellis (100–120 μm)^24^.

## Materials and methods

### Morphological study

Fresh basidiomata were collected during the month of August from different parts of Uttarakhand and Meghalaya. Photographs were taken in the field with a Canon Power Shot SX 50 HS camera. Macromorphological characterizations were done in the field or at basecamp from fresh and dissected basidiomata with the help of daylight. Colour codes and terms mostly follow Kornerup & Wanscher^28^. After noting down all possible macromorphological and macrochemical spot test details, samples were placed for drying in an aluminium field drier. Micromorphological characters were observed after mounting the freehand sections of dried samples in a solution of 5% KOH, 1% Phloxin, and 1% ammoniacal Congo red with an Olympus CX 41 (installed in Central National Herbarium, Botanical Survey of India, Howrah) or Olympus CX 43 compound microscope (installed in Eastern Regional Centre, Botanical Survey of India, Shillong). Drawings of the micromorphological features were made with the help of drawing tube at 1000 × magnification. Microscopic photographs were taken with an Olympus BX 53 or Magcam DC camera. The basidiospores were measured in lateral view. Basidiospore measurements and length/width ratios (Q) are recorded as: minimum–mean–maximum. Basidium length excludes the length of sterigmata. Herbarium codes follow Thiers^29^. Field emission scanning electron microscope (FESEM) illustrations of basidiospores were mounted on a double-sided adhesive tape pasted on a metallic specimen stub and then scanned with a gold coating at different magnifications in high vacuum mode to observe patterns of spore ornamentation. This work was carried out with an FEI Quanta FEG 250 model installed at Centre for Research in Nanoscience and Nanotechnology (CRNN) in University of Calcutta, India.

### DNA extraction, PCR amplification and sequencing

Genomic DNA was extracted from 100 mg of a dried basidioma (for seven species) with the InstaGeneTM Matrix Genomic DNA isolation kit (Biorad, USA) following the manufacturer’s instructions. The PCR amplification of ITS region, part of the LSU, region between conserved domains 6 and 7 of *rpb2* and *tef* 1-α were done using the primer pairs ITS1-F and ITS4; LR0R and LR5; b*rpb2*-6F and f*rpb2*-7cR and ef1-983F and ef1-1567R respectively^30–34^. PCR amplification was carried out in a ProFlex PCR system (Applied Biosystems) programmed for an initial denaturation at 94 °C for 3 min, followed by 35 cycles of denaturation at 94 °C for 1 min, annealing at 50 °C for 30 s, and extension at 72 °C for 1 min. The final extension was kept at 72 °C for 7 min. The PCR products were purified using the QIAquick PCR Purification Kit (QIAGEN, Germany). Both strands of the PCR fragment were sequenced on the ABI 3500 DNA Analyzer (Applied Biosystems, USA) using the amplifying primers. The sequence quality was checked using Sequence Scanner Software ver. 1 (Applied Biosystems). Sequence alignment and required editing of the obtained sequences were carried out using Geneious Pro ver. 5.1^35^. All sequences newly generated in this study were submitted to GenBank. Accession numbers of species used in phylogenetic analysis (Figs. 1, 2, 3, 4, 5) are listed in the Tables 1, 2, 3, 4 and 5.

**Table 1:** *Leccinellum* and allied sequences used in phylogenetic analyses of this study. Newly sequenced collections are in bold.

Species name (as reported in GenBank)	Voucher/strain no.	GenBank accession no.		
		LSU	*rpb2*	*tef* 1-α
*Borofutus dhakanus*	HKAS73789	JQ928616	JQ928597	JQ928576
*Leccinellum albellum*	MICH KUO 07241101	MK601746	–	–
*Leccinellum alborufescens*	FHMU1908	MK816322	MK816333	MK816330
*Leccinellum alborufescens*	FHMU1758	MK816321	MK816332	MK816329
*Leccinellum binderi*	KD 22–015	OQ858379	OQ914387	OR102316
*Leccinellum binderi*	KD 22-007	OQ858380	OQ914386	OR102315
***Leccinellum bothii***	**KD 23-005**	**OR793895**	**OR801234**	**OR801230**
***Leccinellum bothii***	**KD 23-008**	**OR793896**	**OR801235**	**OR801231**
*Leccinellum corsicum*	Buf 4507	KF030347	KF030389	KF030435
*Leccinellum crocipodium*	MICH:KUO-07050707	MK601749	MK766311	MK721103
*Leccinellum crocipodium*	VDKO1006	–	KT824021	KT824054
*Leccinellum fujianense*	FHMU2223	MK816320	MK816336	MK816328
*Leccinellum fujianense*	FHMU2219	MK816319	MK816334	MK816327
*Leccinellum indoaurantiacum*	DC 14-019	KT860059	–	–
*Leccinellum lepidum*	K(M)-142974	MK601751	MK766312	MK721105
*Leccinellum pseudoscabrum*	MICH-60301 R.Watling-6725	MK601754	–	–
*Leccinellum pseudoscabrum*	930808	AF139691	–	–
*Leccinellum pseudoscabrum*	F300	OR602359	–	–
*Leccinellum pseudoscabrum*	CFMR:DPL-11432	MK601752	MK766313	MK721106
***Leccinellum sinoaurantiacum***	**DC ML-52**	**OR786001**	–	**OR801228**
***Leccinellum sinoaurantiacum***	**DC ML-77**	**OR786002**	–	**OR801229**
*Leccinellum sinoaurantiacum*	Li2770	MT154745	MT110428	MT110356
*Leccinellum sinoaurantiacum*	Zang13486	MT154746	–	–
*Leccinellum* sp.	OR0082	–	MZ824749	MZ803024
*Leccinum* aff.* griseum*	KPM-NC-0017381	JN378508	–	JN378449
*Leccinum* aff.* scabrum*	HKAS 57266	KF112442	KF112722	KF112248
*Leccinum album*	Li1072	MW413907	–	MW439267
*Leccinum aurantiacum*	L:0342207	MK601759	MK766318	MK721113
*Leccinum cerinum*	MK11800	AF139692	–	–
*Leccinum duriusculum*	GL4676	AF139699	–	–
*Leccinum duriusculum*	Yang5971	MZ675541	MZ707779	MZ707785
*Leccinum flavostipitatum*	MENMB10801	MH620342		
*Leccinum holopus*	Yang5972	MW413906	MW439258	MW439266
*Leccinum holopus*	9109303	AF139700	–	–
*Leccinum holopus*	MICH: KUO-09150707	MK601763	MK766322	MK721117
*Leccinum manzanitae*	NY-14041 REH-6717	MK601765	MK766324	MK721119
*Leccinum monticola*	HKAS:76669	KF112443	KF112723	KF112249
*Leccinum monticola*	NY-00815448 REH-8591	MK601767	MK766326	MK721121
*Leccinum monticola*	NY-760388 REH-8288	MK601766	MK766325	MK721120
*Leccinum palustre*	MK11107	AF139701	–	–
*Leccinum parascabrum*	Li1700	MW413912	MW439265	MW439272
*Leccinum parascabrum*	Wu1784	MW413911	MW439264	MW439271
*Leccinum pseudoborneense*	WGS965	–	MW439263	––
*Leccinum pseudoborneense*	WGS960	–	MW439262	–
*Leccinum pseudoborneense*	WGS947	MW413908	MW439261	MW439268
*Leccinum quercinum*	HKAS:63502	KF112444	KF112724	KF112250
*Leccinum rugosiceps*	CFMR BOS-866	MK601770	MK766329	MK721124
*Leccinum scabrum*	HKAS56371	KT990587	KT990423	KT990782
*Leccinum scabrum*	KPM-NC-0017840	JN378515	–	JN378455
*Leccinum variicolor*	Lvar1	AF139706	–	
*Leccinum versipelle*	FB27	MZ675546	MZ707782	MZ707790
*Leccinum versipelle*	LJW418	MZ675545	MZ707781	MZ707789
*Leccinum versipelle*	CFMR DLC2002-122	MK601778	MK766336	MK721132
*Octaviania japonimontana*	KPM-NC-0017812	JN378486	–	JN378428
*Octaviania tasmanica*	NY-02449788 REH-10066	MK601798	MK766355	MK721152
*Rossbeevera bispora*	GDGM 45639	MK036347	MK350309	–
*Rossbeevera eucyanea*	KPM-NC0023895	KP222896	–	KP222915
*Rossbeevera griseobrunnea*	GDGM45913	MH537793	–	–
*Rossbeevera griseovelutina*	TNS-F-36991	KC552032	–	KC552077
*Rossbeevera vittatispora*	MEL2321058	KP222895	–	KP222911
*Rossbeevera westraliensis*	OSC61480	JN378505	–	JN378445
*Spongiforma thailandica*	BBH:DED 7873	NG_042464	–	–

**Table 2:** *Phylloporus* and allied sequences used in phylogenetic analyses of this study. Newly sequenced collections are in bold.

Species name (as reported in GenBank)	Voucher no.	GenBank accession no.		
		ITS	LSU	*tef* 1-α
*Phylloporus alboinfuscatus*	N.K. Zeng4179(FHMU3276)	MW588663	MW588626	–
*Phylloporus alboinfuscatus*	JXSB1620	–	MK765818	–
*Phylloporus alborufus*	MAN022	JQ003624	JQ00367	–
*Phylloporus attenuatus*	HKAS:76167	KR094776	KR094780	KR094790
*Phylloporus attenuatus*	HKAS:76168	KR094777	KR094781	KR094791
*Phylloporus bellus*	HKAS 42850	JQ967240	JQ967197	JQ967154
*Phylloporus bogoriensis*	DED7785	JQ003625	JQ003680	–
*Phylloporus brunneiceps*	HKAS 59551	JQ967242	JQ967199	JQ967156
*Phylloporus brunneiceps*	HKAS 56903	NR_120120	NG_042664	JQ967155
*Phylloporus caballeroi*	REH7906	JQ003638	JQ003662	–
*Phylloporus castanopsidis*	MAN104	JQ003642	JQ003689	–
*Phylloporus castanopsidis*	MAN118	JQ003646	JQ003693	–
*Phylloporus catenulatus*	HKAS:76156	KR094774	KR094778	KR094788
*Phylloporus catenulatus*	HKAS:76157	KR094775	KR094779	KR094789
*Phylloporus cyanescens*	REH8681	JQ003621	JQ003684	–
*Phylloporus dimorphus*	MAN128	JQ003648	JQ003697	–
*Phylloporus foliiporus*	JLM1677	JQ003641	JQ003687	–
*Phylloporus gajari*	AG 20-003	OP550185	OP550198	**–**
*Phylloporus gajari*	HKAS:76158	KR231696	KR231697	KR231695
*Phylloporus grossus*	N.K. Zeng3335(FHMU3136)	MW588641	MW588585	MW574464
*Phylloporus grossus*	N.K. Zeng3334(FHMU2937)	MW588640	MW588584	MW574463
***Phylloporus himalayanus***	**KD 23-046**	**OR665532**	**OR663998**	**OR675591**
***Phylloporus himalayanus***	**KD 23-047**	**OR665533**	**OR663999**	**OR675592**
*Phylloporus imbricatus*	HKAS 54,859	JQ967246	JQ967203	JQ967160
*Phylloporus imbricatus*	HKAS 54860	JQ967247	JQ967204	JQ967161
*Phylloporus imbricatus*	HKAS 54861	JQ967248	JQ967205	JQ967162
*Phylloporus imbricatus*	HKAS 54647	JQ967245	JQ967202	JQ967159
*Phylloporus leucomycelinus*	MB00-43	JQ003628	JQ003677	–
*Phylloporus luxiensis*	HKAS 57048	JQ967252	JQ967209	JQ967166
*Phylloporus orientalis*	REH8755	JQ003651	JQ003701	–
*Phylloporus pachycystidiatus*	HKAS 54540	JQ967254	JQ967211	JQ967168
*Phylloporus pelletieri*	Q7199c	JQ003639	JQ003668	–
*Phylloporus pusillus*	OR0484	MH686275	–	MH580802
*Phylloporus pusillus*	OR1310	MH686279	–	MH580804
*Phylloporus rhodoxanthus*	JLM1808	JQ003654	JQ003688	–
*Phylloporus rhodoxanthus*	REH8714	JQ003629	JQ003675	–
*Phylloporus rubeolus*	HKAS 52573	JQ967259	JQ967216	JQ967172
*Phylloporus rubeolus*	HKAS 54543	JQ967261	JQ967218	JQ967174
*Phylloporus rubiginosus*	MAN117	JQ003645	JQ003692	–
*Phylloporus rubrosquamosus*	HKAS 54542	JQ967260	JQ967217	JQ967173
*Phylloporus rufescens*	HKAS 59722	JQ967263	JQ967220	JQ967176
*Phylloporus scabripes*	REH8531	JQ003623	JQ003683	–
***Phylloporus smithii***	**KD 23-012**	**OR656501**	**OR656502**	**OR675589**
***Phylloporus smithii***	**KD 23-022**	**OR656500**	**OR656503**	**OR675590**
*Phylloporus* sp.	HKAS 74679	JQ967271	JQ967228	JQ967184
*Phylloporus* sp.	HKAS 74682	JQ967273	JQ967230	JQ967186
*Phylloporus* sp.	HKAS 74684	JQ967275	JQ967232	JQ967188
*Phylloporus* sp.	HKAS 74688	JQ967279	JQ967236	JQ967191
*Phylloporus* sp.	REH8729	JQ003650	JQ003699	–
*Phylloporus* sp.	OR0989	MH686277	–	MH580811
*Phylloporus* sp.	HKAS 74679	JQ967271	JQ967228	JQ967184
*Phylloporus subbacillisporus*	OR0436	MH686274	–	MH580812
*Phylloporus subrubeolus*	BC022	–	–	MH580813
*Phylloporus subrubeolus*	OR0612	MH686276	–	–
*Phylloporus yunnanensis*	HKAS 52225	JQ967265	JQ967222	JQ967178
*Phylloporus yunnanensis*	HKAS 56999	JQ967267	JQ967224	JQ967180
*Phylloporus yunnanensis*	HKAS 52527	JQ967266	JQ967223	JQ967179
*Phylloporus yunnanensis*	HKAS 58673	JQ967268	JQ967225	JQ967181
*Phylloporus yunnanensis*	HKAS 59412	JQ967269	JQ967226	JQ967182
*Xerocomus magniporus*	HKAS 59820	JQ678697	JQ678699	JQ967195
*Xerocomus subtomentosus*	K 167686	JQ967281	JQ967238	JQ967193

**Table 3:** *Xerocomus* and allied sequences used in phylogenetic analyses of this study. Newly sequenced collections are in bold.

Species name (as reported in GenBank)	Voucher no.	GenBank accession no.	
		ITS	LSU
*Hourangia nigropunctata*	FHMU2230	MT650107	MT650088
*Hourangia nigropunctata*	FHMU2209	MT650106	MT650087
*Hourangia nigropunctata*	FHMU2104	MT650104	MT650085
*Xerocomus albotomentosus*	HKAS90207	–	KT990677
*Xerocomus albotomentosus*	HKAS 74927	–	KF112395
*Xerocomus* cf.* subtomentosus*	JLF2784	KU144808	KU144809
*Xerocomus* cf.* subtomentosus*	JLF2777	KU144806	KU144807
*Xerocomus doodhcha*	KD 13-082	KR611867	KU566806
*Xerocomus ferrugineus*	MICH KUO-08100701	–	MK601820
*Xerocomus ferrugineus*	CFMR BOS-545	–	MK601819
*Xerocomus fraternus*	HKAS 55328	–	NG_059634
*Xerocomus fraternus*	HKAS52526	–	KT990682
*Xerocomus fulvipes*	HKAS52556	–	KT990672
*Xerocomus fulvipes*	HKAS 76666	–	KF112390
*Xerocomus fuscatus*	HKAS53374	–	KT990679
*Xerocomus fuscatus*	JXSB2591	––	MT704383
*Xerocomus fuscatus*	HKAS54753	–	KT990680
*Xerocomus illudens*	MB03-055	–	JQ003705
*Xerocomus illudens*	MB04-016	–	JQ003706
*Xerocomus longistipitatus*	DC 16-056	KY008398	–
*Xerocomus magniporus*	HKAS:59820	JQ678697	JQ678699
*Xerocomus magniporus*	HKAS:58000	KF112392	–
*Xerocomus perplexus*	MB00-005	JQ003657	JQ003702
*Xerocomus puniceiporus*	HKAS 80683	–	KU974141
*Xerocomus reticulostipitatus*	MEH 16_B-7	MF167353	–
*Xerocomus rugosellus*	HKAS68292	–	KT990686
*Xerocomus rugosellus*	HKAS 67749	–	KT990676
***Xerocomus rugosellus***	**KD 23-019**	**OR707912**	**OR707913**
***Xerocomus rugosellus***	**KD 23-055**	**OR707911**	**OR707914**
*Xerocomus silwoodensis*	gs1959	DQ066375	––
*Xerocomus silwoodensis*	MCVE:28973	MH102397	–
*Xerocomus silwoodensis*	AH2005039	DQ438143	–
*Xerocomus subparvus*	HKAS 50295	––	NG_059631
*Xerocomus subparvus*	JXSB1450	–	MK765842
*Xerocomus subparvus*	JXSB1528	–	MK765843
*Xerocomus subtomentosus*	ah1997028	DQ066370	–
*Xerocomus subtomentosus*	K 167686	JQ967281	JQ967238
*Xerocomus subtomentosus*	KM167686	KC215201	KC215222
*Xerocomus uttarakhandae*	KD 22-005	OQ748036	OQ748037
*Xerocomus uttarakhandae*	KD 22-002	OQ748035	OQ74803
*Xerocomus velutinus*	HKAS68135	–	KT990673
*Xerocomus velutinus*	HKAS 52575	–	KF112393
*Xerocomus yunnanensis*	HKAS68282	–	KT990691
*Xerocomus yunnanensis*	HKAS68420	–	KT990690

**Table 4:** *Porphyrellus* and allied sequences used in phylogenetic analyses of this study. Newly sequenced collections are in bold.

Species name (as reported in GenBank)	Voucher no.	GenBank accession no.		
		ITS	LSU	*tef* 1-α
*Afroboletus multijugus*	PC0723571	–	KX869426	KX869299
*Anthracoporus cystidiatus*	HKAS55375	KT990622	MT110410	KT990816
*Anthracoporus cystidiatus*	ZP812	MT154710	–	KT990816
*Anthracoporus holophaeus*	HKAS59407	KT990708	KT990506	KT990888
*Anthracoporus nigropurpureus*	HKAS53370	KT990628	KT990460	KT990822
*Anthracoporus nigropurpureus*	HKAS52685	KT990627	KT990459	KT990821
*Butyriboletus pseudospeciosus*	HKAS63513	KT990541	KT990380	KT990743
*Butyriboletus regius*	KUN-HKAS 84878	MT264910	MT269661	MT269659
*Indoporus shoreae*	AP 6697	MK123976	MK243368	–
*Indoporus shoreae*	AP 6693	MK123973	MK243367	–
*Indoporus squamulosus*	HKAS107153	–	MT110409	MT110335
*Kgaria cyanogranulifera*	REH9207	OR063861	OR263677	–
*Kgaria cyanogranulifera*	REH9196	OR063860	OR263676	–
*Kgaria similis*	REH9031	OR063865	OR263683	–
*Kgaria similis*	REH9033	OR063866	OR263684	–
*Porphyrellus castaneus*	HKAS52554	KT990697	KT990502	KT990883
*Porphyrellus castaneus*	HKAS63076	KT990548	KT990386	KT990749
*Porphyrellus cyaneotinctus*	Hao912	MT154719	–	–
*Porphyrellus cyaneotinctus*	Hao903	MT154718	–	–
*Porphyrellus griseus*	HKAS82849	NG_088126	MT110414	–
*Porphyrellus orientifumosipes*	HKAS84710	MT154717	MT110415	MT110339
*Porphyrellus orientifumosipes*	HKAS53372	KT990629	KT990461	KT990823
*Porphyrellus porphyrosporus*	HKAS:76671	KF112482	KF112718	KF112243
*Porphyrellus porphyrosporus*	MB97-023	DQ534643	GU187800	GU187734
*Porphyrellus pseudocyaneotinctus*	HMJAU 60067	–	OP495792	OP495808
*Porphyrellus pseudocyaneotinctus*	HMJAU 60066	–	OP495791	OP495807
*Porphyrellus pseudocyaneotinctus*	HMJAU 60064	–	OP495789	OP495805
*Porphyrellus pseudocyaneotinctus*	HMJAU 60063	–	OP495788	OP495804
*Porphyrellus pseudocyaneotinctus*	HMJAU 60065	–	OP495790	OP495806
*Porphyrellus pseudocyaneotinctus*	HMJAU 60061	–	OP495786	OP495802
*Porphyrellus pseudocyaneotinctus*	HMJAU 60062	–	OP495787	OP495803
*Porphyrellus pseudocyaneotinctus*	HMJAU 60068	–	OP495793	OP495809
*Porphyrellus scrobiculatus*	HKAS 53366	KF112480	KF112716	KF112241
***Porphyrellus uttarakhandae***	**KD 23-028**	**OR778103**	**OR786650**	**OR801226**
***Porphyrellus uttarakhandae***	**KD 23-056**	**OR778100**	**OR786651**	**OR801227**
*Strobilomyces* sp.	HKAS 59420	KF112463	KF112810	KF112256
*Strobilomyces strobilaceus*	MB001177	–	KX869440	–

**Table 5:** *Retiboletus* and allied sequences used in phylogenetic analyses of this study. Newly sequenced collections are in bold.

Species name (as reported in GenBank)	Voucher no.	GenBank accession no.		
		ITS	LSU	*tef* 1-α
*Pseudoaustroboletus valens*	KUN:HKAS 52603	KM274869	KM274869	KM274877
*Pseudoaustroboletus valens*	KUN:HKAS 82643	–	KM274870	KM274878
*Retiboletus ater*	Li1215	–	MT010611	MT010621
*Retiboletus ater*	Li1224	–	MT010612	MT010622
*Retiboletus ater*	HKAS 56069	OM904960	NG_088116	–
*Retiboletus atrofuscus*	HFJAU10002	–	OL744444	OL963527
*Retiboletus atrofuscus*	HFJAU10003	–	OL744445	OL963526
*Retiboletus brunneolus*	HKAS 52680	OM904973	KF112424	KF112179
*Retiboletus brunneolus*	LC_LJW23	–	MT010615	MT010625
*Retiboletus cyanescens*	KUN-HKAS122940	OM904977	OM904957	ON055268
*Retiboletus cyanescens*	KUN-HKAS106692	OM904978	OM904956	ON055270
*Retiboletus cyanescens*	KUN-HKAS122939	OM904976	OM904954	ON055267
*Retiboletus fuscus*	HKAS74756	–	KT990636	KT990830
*Retiboletus fuscus*	Cui47	–	MT010614	MT010624
*Retiboletus griseus*	BD210	–	HQ161858	–
*Retiboletus griseus*	Halling10162	–	MT010608	MT010618
*Retiboletus griseus*	snBoth	–	KF030308	–
*Retiboletus kauffmanii*	CAL_F_1397	–	KY290586	–
*Retiboletus kauffmanii*	G.Wu352	–	KP739282	KP739301
*Retiboletus kauffmanii*	KUN-HKAS87223	OM904972	OM904945	ON055257
*Retiboletus nigrogriseus*	FHMU2800	–	MH367476	MH367488
*Retiboletus nigrogriseus*	FHMU2045	MH367483	MH367475	MH367487
*Retiboletus ornatipes*	Halling10163	–	MT010617	MT010626
*Retiboletus ornatipes*	Halling10163-1	OM904969	OM904947	ON055261
***Retiboletus pseudoater***	**KD 23-040**	**OR668526**	**OR668533**	**OR683159**
***Retiboletus pseudoater***	**KD 23-048**	**OR668528**	**OR668534**	**OR683160**
*Retiboletus pseudogriseus*	FHMU375	–	MH367477	MH367489
*Retiboletus pseudogriseus*	Zeng647	–	MT154751	MT010623
*Retiboletus retipes*	57/97	–	AF456811	–
*Retiboletus retipes*	116/96	–	AF456823	–
*Retiboletus retipes*	22/97	–	AF456831	–
*Retiboletus retipes*	96/97	–	AF456830	–
*Retiboletus sinensis*	HKAS83957	KP739274	KP739291	KP739303
*Retiboletus sinensis*	HKAS83955	KP739272	KP739289	KP739302
*Retiboletus sinogriseus*	LJ260	–	MT010609	MT010619
*Retiboletus sinogriseus*	LJ258	–	MT010610	MT010620
*Retiboletus vinaceipes*	CFMR:DR-1035 DJL-DR-42	NR_175146	NG_078680	–
*Retiboletus vinaceipes*	CFMR:BZ-2386 BOS-459	MN250217	MN250190	–
*Retiboletus zhangfeii*	HKAS59699	–	JQ928627	JQ928582
*Retiboletus zhangfeii*	HKAS53418	–	KT990630	KT990824

### Alignment and phylogenetic analyses

The ITS, LSU, *rpb2* and *tef* 1-α sequences of the newly generated *Leccinellum bothii*, *L. sinoaurantiacum*, *Phylloporus himalayanus*, *P. smithii*, *Xerocomus rugosellus*, *Porphyrellus uttarakhandae* and *Retiboletus pseudoater* and their close relatives were retrieved from nBLAST search against GenBank (https://www.ncbi.nlm.nih.gov/genbank) and relevant published phylogenies^5,16,18,22–24,36–38^. Four raw datasets (ITS, LSU, *rpb2* and *tef* 1-α) were created separately. All the datasets were aligned separately using the online version of the multiple sequence alignment program MAFFT v. 7 (https://mafft.cbrc.jp/alignment/software/) with L-INS-i strategy and normal alignment mode, respectively. The alignment was checked and trimmed with the conserved motifs manually with MEGA v. 7^39^. To eliminate ambiguously aligned positions in the alignment as objectively as possible, the on-line program Gblocks 0.91b^40^ was used. The program was run with settings allowing for smaller blocks, gaps within these blocks and less strict flanking positions. Species delimitation was first examined using single locus phylogenies. When significant conflict was not observed among the single locus phylogenies, then we concatenated into multi-locus dataset using BioEdit v. 7.0.9^41^. The introns of protein coding genes (*rpb2* and *tef* 1-α) were excluded entirely in the phylogenetic analyses. In the three-locus dataset (LSU + *rpb2* + *tef* 1-α) of *Leccinellum*, 953 bp are for LSU, 770 bp for *rpb2* and 588 bp for *tef* 1-α. In the three-locus dataset (ITS + LSU + *tef* 1-α) of *Phylloporus*, 421 bp are for ITS, 1377 bp for LSU and 602 bp for *tef* 1-α. In the two-locus dataset (ITS + LSU) of *Xerocomus*, 553 bp are for ITS and 840 bp for LSU. In the three-locus dataset (LSU + *rpb2* + *tef* 1-α) of *Porphyrellus*, 880 bp for LSU, 661 bp for *rpb2* and 439 bp for *tef* 1-α. In the three-locus dataset (ITS + LSU + *tef* 1-α) of *Retiboletus*, 550 bp are for ITS, 867 bp for LSU and 582 bp for *tef* 1-α. To find the best-fit evolutionary models of matrixes for IQ-tree and MrBayes were selected using ModelFinder and PartitionFinder 2^42,45^ respectively. The combined dataset was phylogenetically analysed using both maximum likelihood (ML) and Bayesian inference (BI) methods. Maximum likelihood (ML) analysis was conducted using the IQ-tree tool version 2.2.2.6^43^, employing the best model for each locus chosen by ModelFinder^42^. Additionally, ultrafast bootstrap with 1000 replicates was applied to obtain nodal support values. Bayesian inference (BI) was computed in MrBayes v.3.2.6^44^ with four Markov chain Monte Carlo (MCMC) algorithm. PartitionFinder2 was used to find the best nucleotide substitution models using the Bayesian information criterion (BIC) with a greedy search over all models^45^. Two MCMC runs of four chains were executed simultaneously from a random starting tree for 100,000 generations until the standard deviation of split frequencies reached below the 0.01 threshold. Trees were sampled every 100th generation. The first 25% of trees were discarded as burn-in. Chain convergence was determined using Tracer 1.5^46^ to ensure sufficiently large effective sample size (ESS) values (> 200). Gaps in the alignment were treated as missing data in phylogenetic analyses. Both ML and BI analyses resulted in essentially the same tree topologies and our five novel taxa are presented in the phylogenetic trees in bold red font (Figs. 1, 2, 3, 4, 5). Maximum likelihood bootstrap (MLbs) values ≥ 70% and Bayesian posterior probabilities (BPP) values ≥ 0.95 are shown in the phylogenetic trees.

### Statements

The present research was undertaken in India, and the authors have obtained all kinds of permission or licences for the respective macrofungal surveys and collections of wild mushrooms for research purpose. Voucher specimens were duly submitted in the public herbaria: CAL and ASSAM (both are indexed in Index Herbariorum, https://sweetgum.nybg.org/science/ih/). The authors herewith confirm that all field studies and corresponding collections of mushrooms are complied with relevant institutional, national, and international guidelines and legislation.

## Discussion

Boletaceae, the fastest revealing family among mushroom forming ectomycorrhizal Basidiomycota is now comprising of over 100 genera that are only came into the light with the combined approach of multigene molecular phylogeny and morphology. Considerable studies have been undertaken across the continents especially during last one decade and this family has undergone dramatic taxonomic reassessment. Several novel genera and numerous novel species are continually being uncovered across the continents in general and Asian countries in particular. Only in past five to six years, about 22 genera were discovered in this family from all over the world namely, *Acyanoboletus* G. Wu & Zhu L. Yang, *Afrocastellanoa* M.E. Smith & Orihara, *Brasilioporus* A.C. Magnago, Alves-Silva & T.W Henkel, *Cacaoporus* Raspé & Vadthanarat, *Carolinigaster* M.E. Sm. & S. Cruz, *Erythrophylloporus* Ming Zhang & T.H. Li, *Hemiaustroboletus* Ayala-Vásquez, García-Jiménez & Garibay-Orijel, *Hemilanmaoa* Yang Wang, Bo Zhang & Y. Li, *Hongoboletus* G. Wu & Zhu L. Yang, *Indoporus* A. Parihar, K. Das, Hembrom & Vizzini, *Ionosporus* O. Khmelnitsky, *Kaziboletus* Hosen & Zhu L. Yang, *Kgaria* Halling, Fechner & Davoodian, *Longistriata* Sulzbacher, Orihara, Grebenc, M.P. Martín & Baseia, *Neotropicomus* A.C. Magnago, Alves-Silva & T.W Henkel, *Nevesoporus* A.C. Magnago & T.W. Henkel, *Phylloporopsis* Angelini, A. Farid, Gelardi, M.E. Smith, Costanzo, & Vizzini, *Rostrupomyces* Vadthanarat & Raspé, *Rubinosporus* Vadthanarat, Raspé & Lumyong, *Spongispora* G. Wu, S.M.L. Lee, E. Horak & Zhu L. Yang, *Tropicoboletus* Angelini, Gelardi & Vizzini and *Villoboletus* L. Fan & N. Mao^37,38,47–63^. It is noteworthy that 11 out of 22 genera are established from Asian countries like, China, Thailand and India. But unlike China and Thailand the megadiverse country like India remains seriously under-focussed in terms of Boletaceae. The stretch of Indian Himalaya and surrounding hilly regions are the hub for the ectomycorrhizal mushrooms including Boletaceae. Except few sporadic works there was no systematic documentary. Therefore, Indian taxa are remained unattended, uncovered or undiscovered. There is a high chance that considerable numbers of these taxa will be extinct due to uncontrolled man-made activities even before they are discovered. Exploration by the trained mushroom-taxonomists and the documentation would be the only hope to create the awareness and save these creatures, Moreover, most of the Indian elements are wrongly known by their European or North American lookalikes. Keeping in view the number of unaddressed species in different forests of Indian Himalaya and its adjacent hills, scarcity of boletologists, only in 2022 the project on boletoid mushrooms were proposed for the first time by Botanical Survey of India, premier research institute of Ministry of Environment, Forest and Climate Change (Govt. of India). Our extensive and intensive survey followed by methodical morphology-based characterization, molecular phylogenetic estimation and documentation will not only open the avenues for research on boletoid mushrooms of India but also resolve the many hidden mystery.

## Data Availability

The authors also state that all the raw data of this research and findings are available from the first author (K.D.).
